# Nisin-Loaded Solid Lipid Nanoparticles (SLN-Nisin) Mitigate *Treponema denticola* Intracellular Infection and Restore Highly Dysregulated Host Gene Expression

**DOI:** 10.3390/ph19101585

**Published:** 2026-10-07

**Authors:** Allan Radaic, Erin Malone, Shen Lin, Lisa Danielly Curcino Araujo, Ivan Tse, Sean Ganther, Pachiyappan Kamarajan, J. Christopher Fenno, Yvonne L. Hernandez-Kapila

**Affiliations:** 1 School of Dentistry, University of California, Los Angeles (UCLA), Los Angeles, CA 90095, USA; aradaic@dentistry.ucla.edu (A.R.);; 2 School of Dentistry, University of California, San Francisco (UCSF), San Francisco, CA 94143, USA; 3 School of Dentistry of Ribeirao Preto, University of São Paulo, Ribeirao Preto, SP 14040-904, Brazil; 4 School of Dentistry, University of Michigan, Ann Arbor, MI 48109, USA; fenno@umich.edu

**Keywords:** periodontal disease, *Treponema denticola*, nisin, solid lipid nanoparticles, SLN-Nisin, antimicrobial peptides

## Abstract

**Background/Objectives:** Periodontal disease is a bacterially driven chronic inflammatory condition that compromises tooth-supporting tissues. *Treponema denticola* is strongly associates with periodontal tissue destruction and tooth loss. Given the contribution of antibiotic overuse, the rise of multi-drug resistance represents a major therapeutic challenge. Previous literature has shown that nisin, an antimicrobial peptide, exhibits activity against periodontal pathobionts, including *T. denticola*. Yet, nanoparticle-based delivery systems, such as solid lipid nanoparticles (SLNs), may enhance nisin’s intracellular activity and therapeutic potential. Therefore, the objective of this work was to evaluate the therapeutic potential of SLN-Nisin in treating intracellular *T. denticola* infection in human periodontal ligament cells in periodontal disease in vitro settings. **Methods:** SLNs and SLN-Nisin were prepared by microemulsion extrusion. *T. denticola* intracellular trafficking in human-derived periodontal ligament (hPDL) cells was assessed by confocal microscopy using endocytosis fluorescent markers. RNA-seq characterized *T. denticola*-induced transcriptional alterations. SLN-Nisin intracellular antimicrobial activity was assessed by confocal microscopy and bacterial viability assays, while mitigation of host transcriptional alterations by RT-qPCR. **Results**: *T. denticola* invades hPDL cells and undergoes dynamic intracellular trafficking through the endocytic pathway, resulting in sustained late endosomal colonization, promoting significant host transcriptional alterations. Further, SLN-Nisin is efficiently internalized by hPDL cells through clathrin-mediated endocytosis and phagocytosis, and both nisin and SLN-Nisin significantly reduce bacterial burden. SLN-Nisin restores four of the most strongly dysregulated host genes to control levels, whereas nisin restores only two. **Conclusions**: These findings demonstrate that SLN-based delivery enhances the intracellular efficacy of nisin, limiting *T. denticola* burden and reversing infection-induced host transcriptional alterations. SLN-Nisin represents a promising therapeutic approach for targeting intracellular *T. denticola* infection in periodontal disease.

## 1. Introduction

Periodontal disease, such as gingivitis and periodontitis, is a bacterially-driven chronic oral inflammatory disease that compromises the integrity of the tooth-supporting tissues, including the gingiva, periodontal ligament and alveolar bone [1,2]. The 2009–2014 National Health and Nutrition Examination Survey (NHANES) report indicates that 42.2% of US adults 30 years or older have periodontitis, with almost a fifth of those considered severe periodontitis [3]. Globally, results from the 2019 Global Burden of Diseases indicate the prevalence of periodontitis doubled from 1990 to 2019 [4]. Additionally, it has been estimated that the disease treatment costs are over $400 billion US dollars per year and over $50 billion US dollars are spent per year in loss of productivity [5]. There is a strong interplay between periodontal disease and other systemic diseases, such as diabetes, cardiovascular diseases, oral cancer, pre-term birth and Alzheimer’s disease [6,7,8,9], which could inflate these figures even further.

Periodontal disease etiology has been extensively studied, with the most accepted hypothesis being that of the polymicrobial synergy and dysbiosis model, in which several low-abundance microbes synergistically promote a remodeling of the oral microbiome that subvert and modulate the host’s defenses [1,10]. Among the identified species in this process, *Treponema denticola*, a Gram negative oral spirochete, has been implicated in the advanced or aggressive forms of the disease and in periodontal disease recurrence [11]. *T. denticola* also mediates cytoskeletal and extracellular breakdown and the dysregulation of tissue destructive genes in human-derived periodontal ligament (hPDL) cells [11,12].

Interestingly, these microbes are known to undergo internalization into host cells and thereby avoid the actions of antimicrobials and the host immune response, causing persistent and recurrent infections [13,14]. In fact, *T. denticola* can invade human gingival epithelial cells [15,16] and hPDL cells [17] via specific cell surface receptors, including TLR2 [17]. This fact, associated with the current crisis of antimicrobial resistance and the unavailability of new antibiotics, underscores the need for novel antimicrobial approaches to address these global issues. Antimicrobial peptides (AMPs) are emerging antimicrobial agents produced by several bacterial phyla, plants, animals, and even humans [1,18]. These peptides are known for their broad spectrum of action, i.e., active against both Gram-positive and -negative bacteria [18]. Although most AMPs demonstrate antimicrobial activity due to cellular membrane damage, some can also target certain proteins, as well as their target species’ DNA and RNA, making them highly suitable as replacements for classical antibiotics [1,18].

In light of this, nisin, an antimicrobial peptide currently used in food preservation, is an effective periodontopathic bacteria inhibitor [17]. Mechanistically, it has been reported that nisin blocks *T. denticola* internalization and pathogen-triggered Matrix Metalloproteinase-2 (MMP-2) activation via TLR2 [17]. Given the potential use of nisin in the management of periodontal disease and periodontal disease-associated systemic diseases, optimization and/or enhancement of nisin’s capabilities through nano-sized drug delivery systems (nano-DDSs) warrant consideration.

Nano-DDSs are devices or formulations in the nanometric range that modify and improve a therapeutic agent’s efficacy and safety by controlling the rate, time, and/or selectivity of drug release in the body [19,20]. Additionally, nano-DDSs can be designed to modify the pharmacokinetics and biodistribution of therapeutic agents, to protect agents from degradative enzymes, and to act as drug reservoirs in the targeted tissue [21]. Among the currently available nano-DDS platforms, solid lipid nanoparticles (SLNs) represent one of the promising candidates. In particular, SLNs are a type of lipid nanoparticle (LNP) composed of biocompatible lipid core that remain solid at both room and physiological temperatures and is stabilized by at least one surfactant layer, exhibiting long-term physical–chemical stability and potential for scalable production [22,23,24,25]. Our group previously developed an SLN-based formulation of nisin (SLN-Nisin), which showed significantly greater activity in suppressing planktonic *T. denticola* growth, and disrupting human-derived oral biofilms, compared to free nisin in vitro [25].

**Figure 1:**
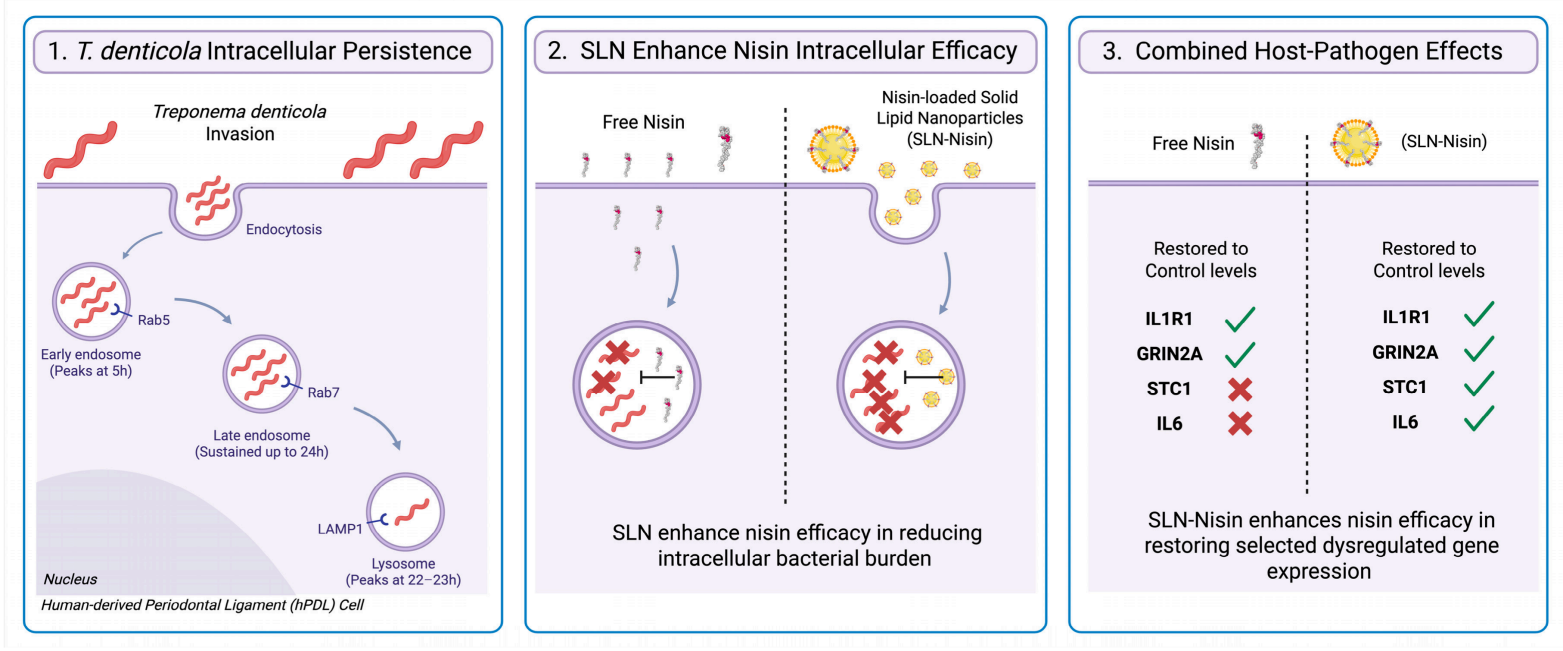
Schematic overview of three key aspects of the intracellular activity of SLN-Nisin against *T. denticola* presented in this study. (Panel 1) Following internalization into human periodontal ligament (hPDL) cells, *T. denticola* traffics through early and late endosomal compartments and subsequently to lysosomes. A viable *T. denticola* population persists within late endosomes for up to 24 h, highlighting a potential mechanism by which the pathogen may evade immune surveillance and antimicrobial agents. (Panel 2) SLN-mediated delivery enhances nisin activity against intracellular *T. denticola*. Depending on the experimental readout, SLN-Nisin produced effects ranging from a reduction comparable to that of free nisin to the complete elimination of detectable intracellular bacterial signal, whereas free nisin produced only a partial reduction at the same nisin-equivalent concentration. (Panel 3) SLN-Nisin also enhances the recovery of selected *T. denticola*-dysregulated host gene expression toward control levels. Thus, SLN-Nisin effects extend beyond reducing the intracellular bacterial burden to promoting the recovery of host-cell homeostasis, potentially shifting the current therapeutic paradigm toward the combined control of infection and restoration of normal tissue physiology.

Therefore, the objective of this work was to evaluate the therapeutic potential of SLN-Nisin in treating intracellular *T. denticola* infection in human cells in in vitro settings with relevance to periodontal disease (Figure 1).

## 2. Results

To examine the therapeutic potential of SLN-Nisin in treating intracellular *T. denticola*, we performed a series of cell internalization experiments with the bacterium to further understand its invasion pathway.

**Figure 2:**
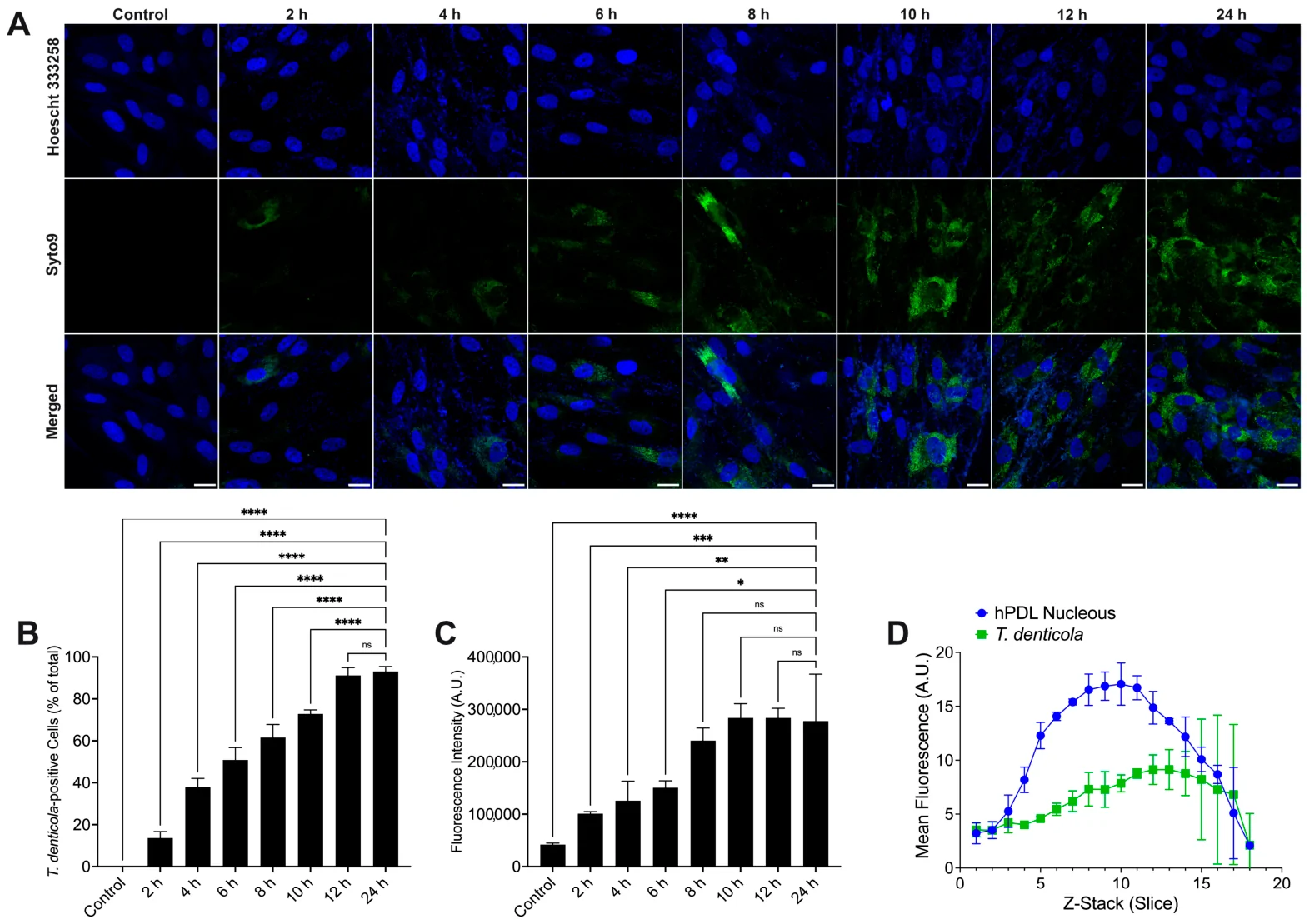
*T. denticola* is significantly internalized by hPDL cells, saturating at 12 h after infection. (**A**) Confocal microscopy of hPDL nuclei (Hoescht 333258; blue) and *T. denticola* (Syto9; green) and the merged image (blue and green). Columns indicate the time points from 0 h (Control; left) to 24 h (right). (**B**) Semi-quantification of the images expressed as percentage of *T. denticola* (Syto9)-positive cells over time. (**C**) Semi-quantification of the images using *T. denticola* fluorescence (Syto9) intensity over time. (**D**) Mean fluorescence intensity in each Z-Stack slice of Hoescht 333258-stained hPDL cell nuclei (blue) and Syto9-stained *T. denticola* (green) after 24 h of infection. “ns” means not significant between marked samples, * means *p* ≤ 0.05 between marked samples, ** means *p* ≤ 0.01 between marked samples, *** means *p* ≤ 0.001 between marked samples and **** means *p* ≤ 0.0001 between marked samples. The scale bar represents 20 µm. Data were obtained from three independent biological replicates (separate cell passages), each analyzed in triplicate.

We started this study by investigating *T. denticola* invasion kinetics (Figure 2). The results showed increasing amounts of *T. denticola* (Syto9; Green) invading the hPDL (Hoescht 333258; Blue) cells over time (Figure 2A). Syto9 is a fluorescent nucleic acid stain, enabling the visualization of *T. denticola* without impairing the pathogen function or their viability [26]. The percentage of *T. denticola*-positive hPDL cells significantly increased over time and stabilized at 12 h. To further characterize the temporal dynamics of invasion, the 24 h time point was used as the statistical reference to evaluate potential saturation of the invasion process. No significant difference was observed at 12 h, indicating potential saturation in the number of *T. denticola*-positive cells at the time point compared to 24 h (Figure 2B). Evaluation of *T. denticola* fluorescence intensity, as a measure of intracellular bacterial burden, defined here as the amount of *T. denticola* detected within hPDL cells, revealed a significant time-dependent increase that reached a plateau by 8 h of infection, with no significant differences observed among the 8, 10, 12, and 24 h time points (Figure 2C).

Given these results, Z-stacks were taken after 24 h of invasion to evaluate whether *T. denticola* bacteria indeed invaded the hPDL cells or whether *T. denticola* was merely bound to the surface of the hPDL cells. 3D reconstruction of the Z-stacks (Movies S1 and S2) and analysis of the hPDL cell nuclei and *T. denticola* genetic material (nucleic acid content) fluorescence intensity slice by slice (Figure 2D) indicates that *T. denticola* was indeed located intracellularly in the same plane as the hPDL cell nuclei.

**Figure 3:**
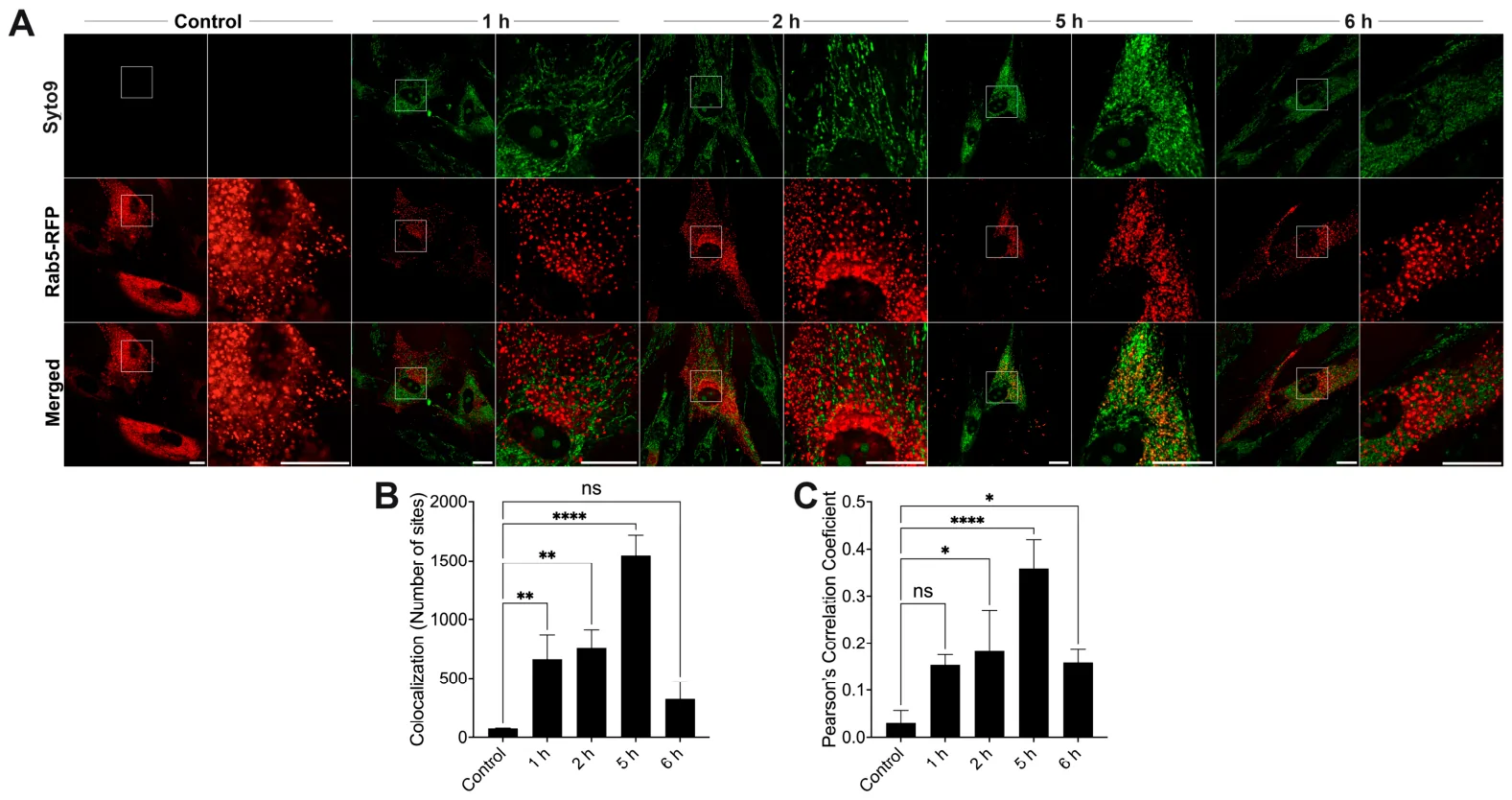
*T. denticola* colocalizes with Rab5-positive early endosomes, peaking at 5 h of infection. (**A**) Confocal microscopy of *T. denticola* (Syto9; green) and hPDL (Rab5; red) and the merged image (green, and red). Columns indicate the time points from 0 h (Control; left) to 24 h (right), as well as uncropped (63× magnification; left column in the same time point) and cropped/zoomed in (right column in the same time point) images of each time point for more clarity. Colocalizations between *T. denticola* (green; Syto9) and Rab5-RFP (red) by (**B**) number of sites, determined using object-based analysis, and (**C**) Pearson’s correlation coefficient using the JaCoP plugin in Fiji/ImageJ, as described in the Methods. “ns” means not significant between marked samples, * means *p* ≤ 0.05 between marked samples, ** means *p* ≤ 0.01 between marked samples, and **** means *p* ≤ 0.0001 between marked samples. The scale bar represents 20 µm. Data were obtained from three independent biological replicates (separate cell passages), each analyzed in triplicate.

Considering previous literature showing endocytosis-based invasion of *T. denticola* in human gingival epithelial cells [15,16], we hypothesized that the bacterium would invade hPDL cells using a similar mechanism. To test this, Syto9-stained *T. denticola* were assessed for colocalization with RFP-tagged Rab5, an early endosome marker [27] (Figure 3). At the end of each time point, gentamycin was used to eliminate any extracellular bacteria. Object-based colocalization analysis was used to assess the spatial association between *T. denticola* and Rab5. Following intensity thresholding to distinguish fluorescent objects from background, objects in the two channels were considered colocalized when the distance between their fluorescence intensity centers in the x, y plane was below the microscope’s optical resolution [28]. This analysis revealed that *T. denticola* colocalization with Rab5 peaked at 5 h of infection, reaching 1548.0 ± 165.0 colocalization sites (Figure 3B).

The results indicate that *T. denticola* colocalized with Rab5 over time, peaking at 5 h of infection as assessed by detection of 1548.0 ± 165.0 colocalization sites (Figure 3B).

Pearson’s correlation coefficient was also used to quantify the degree of correlation between the fluorescence intensities of the green (Syto9-*T. denticola*) and the red (Rab5-RFP) channels. Pearson’s correlation coefficient ranges from −1 to +1. Positive values indicate an increasing linearly relationship between the two channel fluorescence intensities, with +1 values representing a perfect linear relationship (i.e., a complete colocalization). Negative values, on the other hand, indicate an inverse relationship between the channels, with −1 representing perfect negative or inverse correlation to one another. A value of 0 indicates no linear correlation between the two fluorescence channels [28,29]. Consistent with the peak in the number of colocalization sites, Pearson’s correlation analysis showed the strongest correlation between *T. denticola* and Rab5 fluorescence signals at 5 h of infection (0.36 ± 0.06; Figure 3C).

At 6 h, colocalization sharply decreased to 326.0 ± 142.8 colocalization sites and a Pearson’s correlation of 0.16 ± 0.03 at 6 h of infection, indicating progression of the bacteria through the endocytosis pathway.

**Figure 4:**
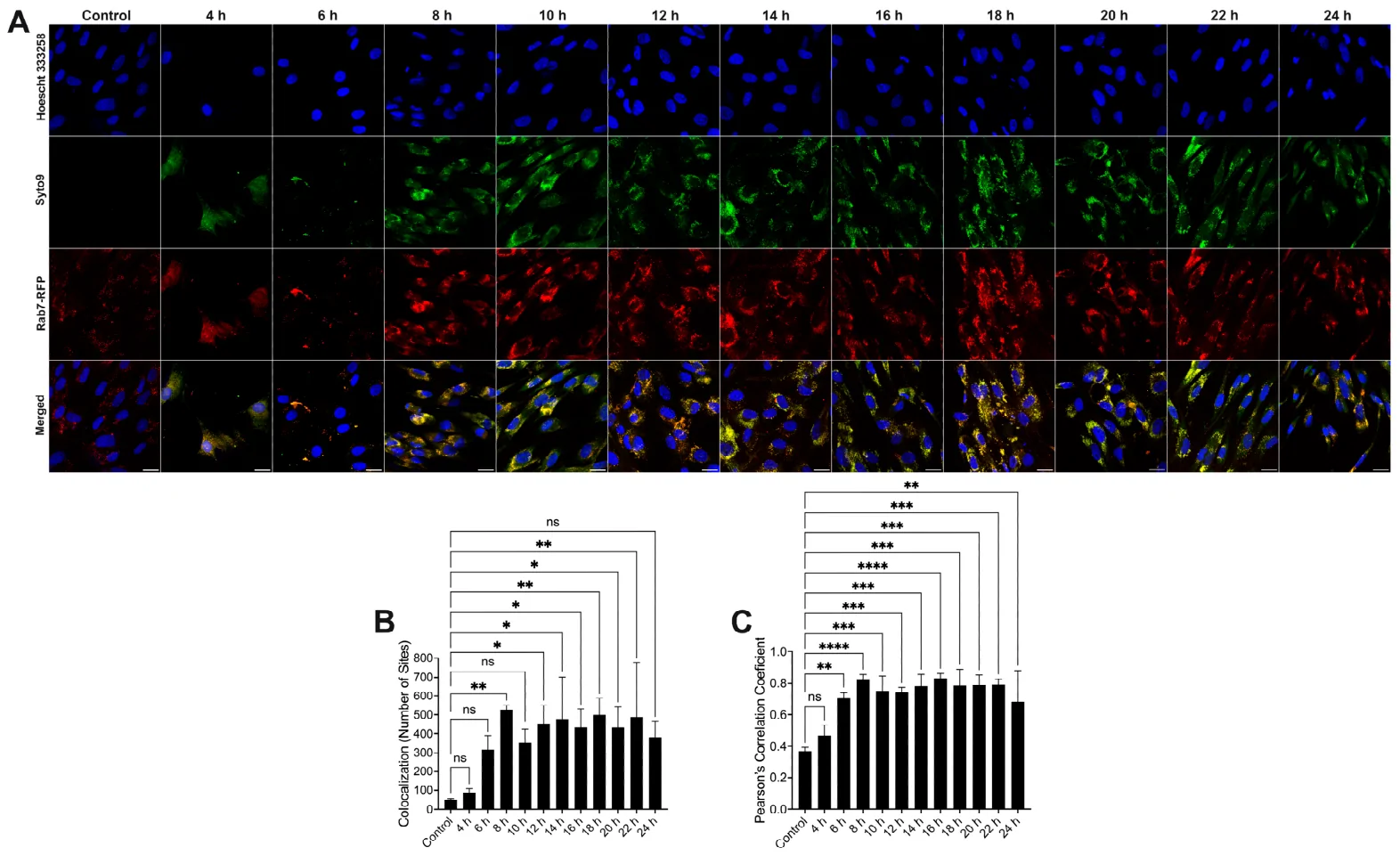
Persistent colocalization of *T. denticola* with Rab7-positive late endosome from 8 to 24 h of infection. (**A**) Confocal microscopy of hPDL cells (Hoescht 333258, blue) infected by *T. denticola* (Syto9; green); Rab7-RFP (red) and the merged image (blue, green, and red). Columns indicate the time points from control (left) up to 24 h (right). Colocalizations between *T. denticola* (Syto9) and Rab7-RFP by (**B**) number of sites and (**C**) Pearson’s correlation coefficient. “ns” means not significant between marked samples, * means *p* ≤ 0.05 between marked samples, ** means *p* ≤ 0.01 between marked samples, *** means *p* ≤ 0.001 between marked samples, **** means *p* ≤ 0.0001 between marked samples. The scale bar represents 20 µm. Data were obtained from three independent biological replicates (separate cell passages), each analyzed in triplicate.

Next, trafficking to late endosomes was similarly evaluated using RFP-tagging Rab7, a late endosome marker [30,31] (Figure 4). At the end of each time point, gentamycin was used to eliminate any extracellular bacteria. The results indicate increasing colocalization of *T. denticola* with the late endosome from 4 h up to 8 h, followed by significant sustained colocalization compared with the control. The number of colocalization sites ranged between 349 ± 139.7 (at 24 h) and 518 ± 31.1 (at 8 h) and Pearson’s coefficient ranged from 0.68 ± 0.20 (at 24 h) to 0.834 ± 0.02 (at 16 h). These data indicate potential translocation of the bacterium from the early endosome to the late endosome (between 4 h and 8 h), followed by intracellular persistence of *T. denticola*.

**Figure 5:**
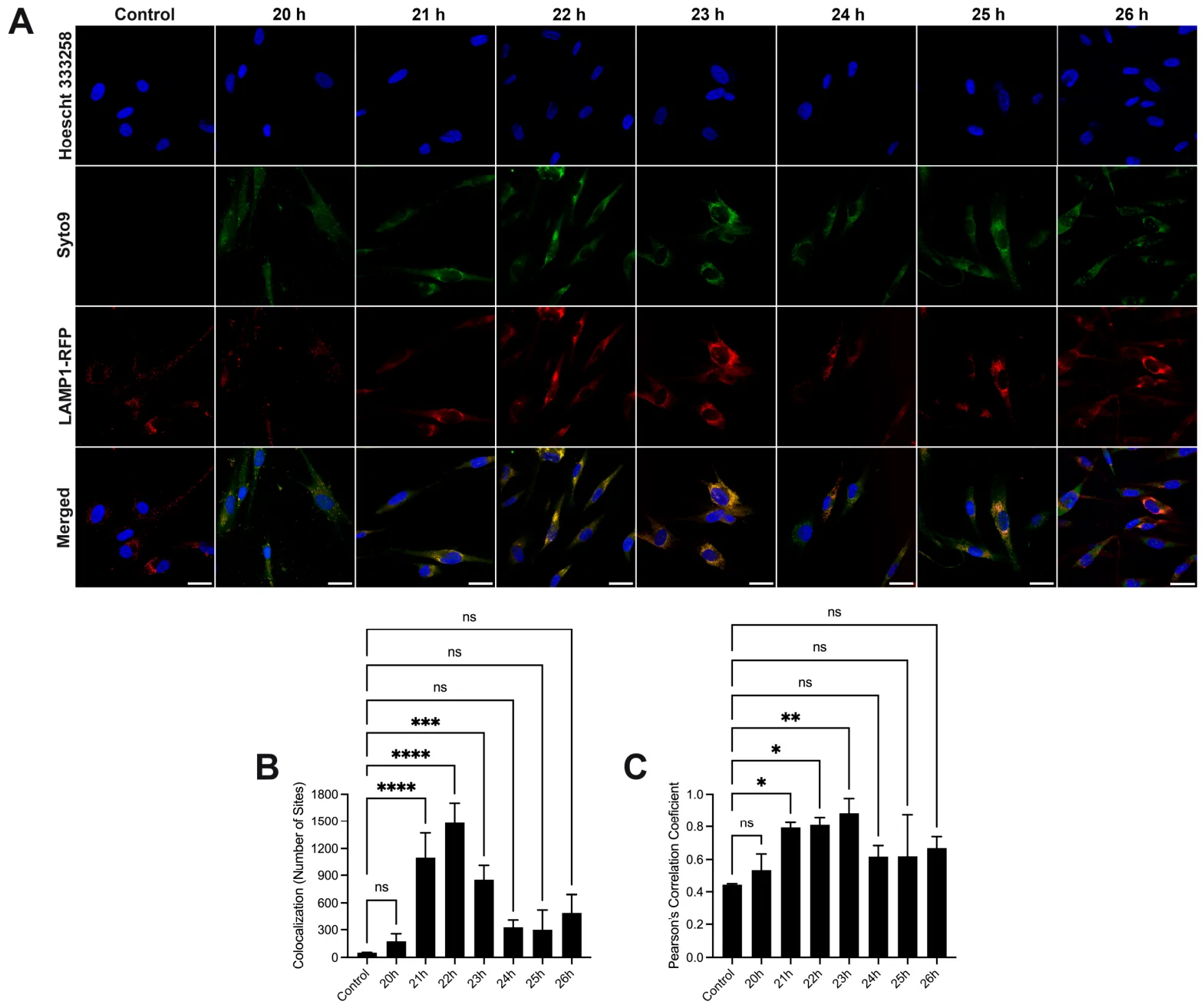
*T. denticola* colocalizes with lysosomal marker LAMP1, peaking at 22 h of infection. (**A**) Confocal microscopy of hPDL cells (Hoescht 333258, blue) infected by *T. denticola* (Syto9, green); LAMP1-RFP (red) and the merged image (blue, green, and red). Columns indicate the time points from control (left) up to 26 h (right). Colocalizations between *T. denticola* (Syto9) and LAMP1-RFP by (**B**) number of sites and (**C**) Pearson’s correlation coefficient. “ns” means not significant between marked samples, * means *p* ≤ 0.05 between marked samples, ** means *p* ≤ 0.01 between marked samples, *** means *p* ≤ 0.001 between marked samples and **** means *p* ≤ 0.0001 between marked samples. The scale bar represents 20 µm. Data were obtained from three independent biological replicates (separate cell passages), each analyzed in triplicate.

Lastly in this series of experiments, we investigated the trafficking of *T. denticola* to lysosomes, the final compartment in the endocytosis pathway. Similar to the previous experiments, LAMP1, a lysosomal marker [32] was RFP-tagged and bacterial localization was assessed using Syto9-stained *T. denticola* (Figure 5). At the end of each time point, gentamycin was used to eliminate any extracellular bacteria. The results indicate significant *T. denticola* colocalization with lysosomes between 21 h and 23 h following infection, compared to control. Colocalization sites peaked at 22 h (1481 ± 220.6) and there was a Pearson’s correlation coefficient of 0.8843 ± 0.09 at 23 h. This peak value was followed by a sharp decrease in the number of colocalization sites (to 299 ± 223.4 sites) and Pearson’s correlation (0.61 ± 0.06); not significantly different than the control and indicating a potential end to the lysosomal processing at 24 h.

Taken together, the data indicate progressive trafficking of intracellular *T. denticola* into late endosomal and lysosomal compartments, where *T. denticola* may be undergoing dynamic, time-dependent intracellular processing. A subpopulation of bacteria persisted in late endosomal compartments while another subset was delivered and processed by the lysosomes at 24 h, indicating differential processing dynamics within intracellular compartments.

Having established the intracellular invasion and trafficking of *T. denticola* within the endocytosis pathway, we next investigated the degree to which SLN-Nisin might inhibit *T. denticola* burden. To achieve this, we first examined SLN-Nisin’s ability to internalize into hPDL cells. We observed that 90% of the cells internalized the nanoparticles within 1 h (Figure S1A) and nanoparticle fluorescence intensity peaked at 4 h (Figure S1B) The internalization mechanism was primarily mediated via phagocytosis and clathrin-mediated endocytosis (Figure S1C,D), since chemical inhibitors to these processes abrogated the internalization.

Next, we evaluated the potential for nisin and SLN-Nisin to inhibit *T. denticola* burden after 24 h of exposure. The 24 h infection time point was selected because intracellular trafficking analysis demonstrated that *T. denticola* had fully established infection within host cells, characterized by predominant localization in late endosomes and completion of lysosomal trafficking by this stage, reflecting a sustained intracellular infection state.

At the end of treatment, gentamycin was used to eliminate any extracellular bacteria. We used flow cytometry (Figure S2) as a preliminary assessment of the effects of nisin and SLN-Nisin on *T. denticola* burden. We found that both treatments significantly reduced *T. denticola* fluorescence intensity after 4 h of treatment (Figure S2B) compared to the infected control. No significant differences were observed in the percentage of *T. denticola*-positive hPDL cells at this early time point (Figure S2A).

**Figure 6:**
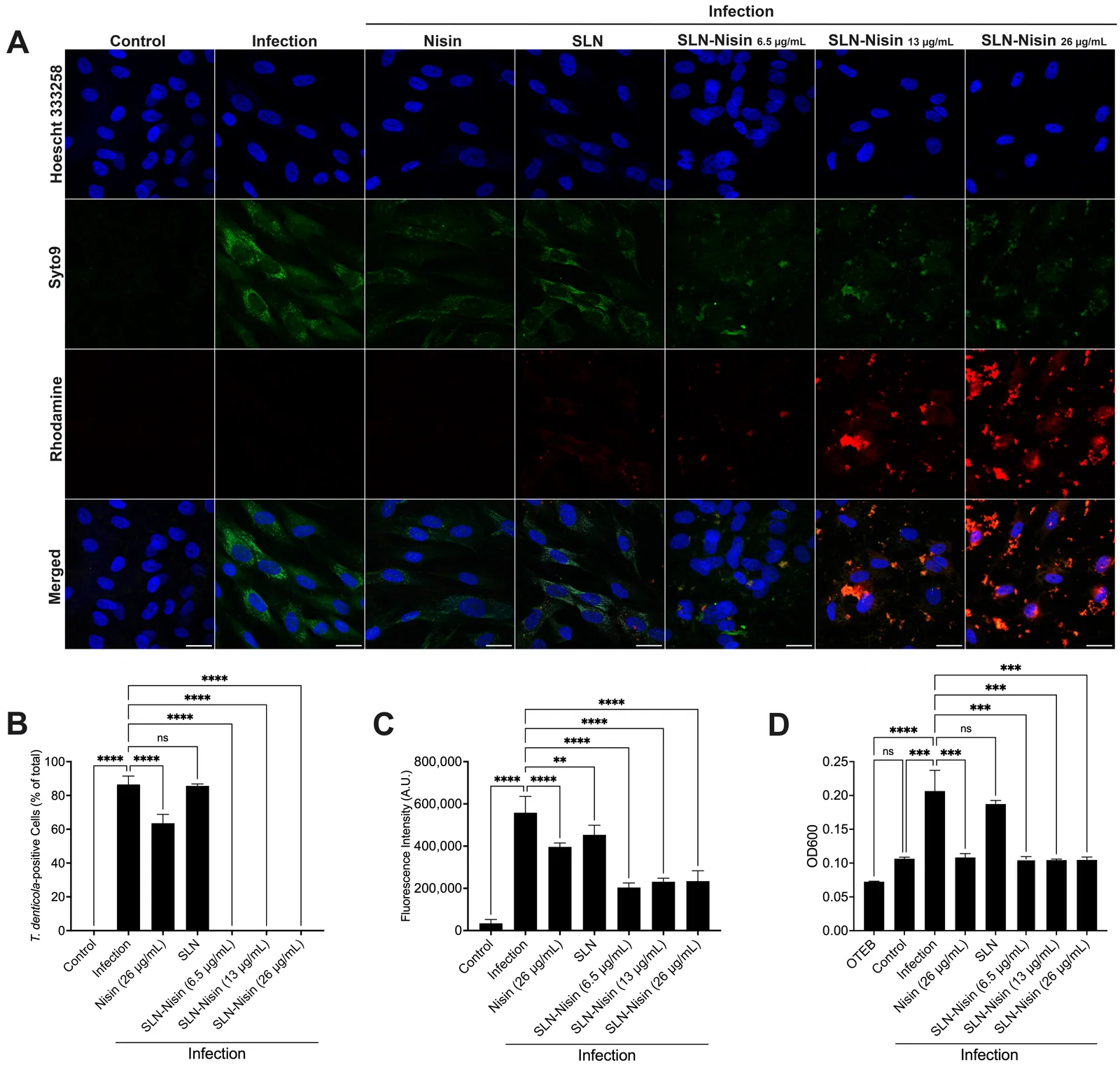
Nisin and SLN-Nisin significantly reduces *T. denticola* burden and intracellular viability in hPDL cells. (**A**) Confocal microscopy of hPDL cells (Hoescht 333258, blue) infected by *T. denticola* (Syto9, green). SLN and SLN-Nisin were fluorescently labeled with rhodamine (red) to visualize their cellular uptake and intracellular distribution. Merged images show the blue, green, and red fluorescence channels. Columns indicate treatments. (**B**) Semi-quantification of the images expressed as percentage of *T. denticola* (Syto9)-positive cells. (**C**) Semi-quantification of the images expressed as *T. denticola* fluorescence (Syto9) intensity. (**D**) *T. denticola* Intracellular viability/regrowth in hPDL cells treated with either nisin or SLN-Nisin. “ns” means not significant between marked samples, ** means *p* ≤ 0.01 between marked samples. *** means *p* ≤ 0.001 between marked samples. **** means *p* ≤ 0.0001 between marked samples. The scale bar represents 20 µm. Data were obtained from three independent biological replicates (separate cell passages), each analyzed in triplicate.

Building upon this data, confocal microscopy was performed to determine the full extent to which nisin and SLN-Nisin limit intracellular bacterial burden (Figure 6). To evaluate the therapeutic effects of nisin and SLN-Nisin, statistical comparisons were performed relative to the *T. denticola* infection. This approach was selected to determine whether each treatment significantly reduced intracellular bacterial burden, thereby directly assessing its ability to mitigate the effects of established *T. denticola* intracellular burden.

The analysis demonstrated that at 24 h, 86.5 ± 5.0% of the hPDL were positively infected with *T. denticola* (infection group) and showing a fluorescence intensity of 558,092 ± 77,399. Nisin (26 µg/mL) significantly decreased the positivity to 63.6 ± 5.3% of the cells and an intensity of 396,497 ± 18,243; internalized *T. denticola* puncta can still be seen present in the cells. All three SLN-Nisin concentrations significantly eliminated (0% positivity) all the intracellular puncta and significantly decreased the intensity fluorescence to 203,810 ± 21,624, 231,160 ± 17,015 and 234,336 ± 48,975. Given the seemingly total elimination of intracellular puncta, it is possible that the fluorescence seen is likely background from the bacterial genetic material cell remnants degraded by the hPDL cells.

Interestingly, we observed that both *T. denticola* and SLN-Nisin colocalize in the late endosome (Figure S3), suggesting that nanoparticle-mediated delivery may facilitate direct interaction between nisin and intracellular *T. denticola* within host cells.

Given these results, we hypothesized that *T. denticola* remained viable following 24 h of intracellular residence in hPDL cells, while SLN-Nisin compromised bacterial viability following 24 h of treatment. To assess this, gentamycin was used to eliminate any extracellular bacteria at the end of treatment and water was added to osmotically burst the hPDL cells. The cells remnants were collected and grown in OTEB media under anaerobic conditions to determine whether the internalized *T. denticola* remained viable. The results revealed (Figure 6D) a significant increase in the media absorbance in the *T. denticola* infection group, compared to no-infection control and free OTEB media group, suggesting that *T. denticola* indeed remained viable inside the cells after 24 h of invasion. In fact, we observed the presence of *T. denticola* puncta inside hPDL cells 48 h and 96 h after infection (Figures S4 and S5, respectively), indicating that *T. denticola* may remain viable even later than 24 h.

Both nisin (26 µg/mL) and SLN-Nisin (6.5, 13 and 26 µg/mL) were able to significantly reduce *T. denticola* intracellular viability/regrowth capabilities. SLNs alone showed no significant difference compared to *T. denticola* infection. These results indicate that incorporating nisin into SLNs doubles the bacteriocin efficacy in reducing *T. denticola* intracellular viability, as SLN-Nisin at 13 µg/mL presented similar results as compared to a nisin concentration of 26 µg/mL.

**Figure 7:**
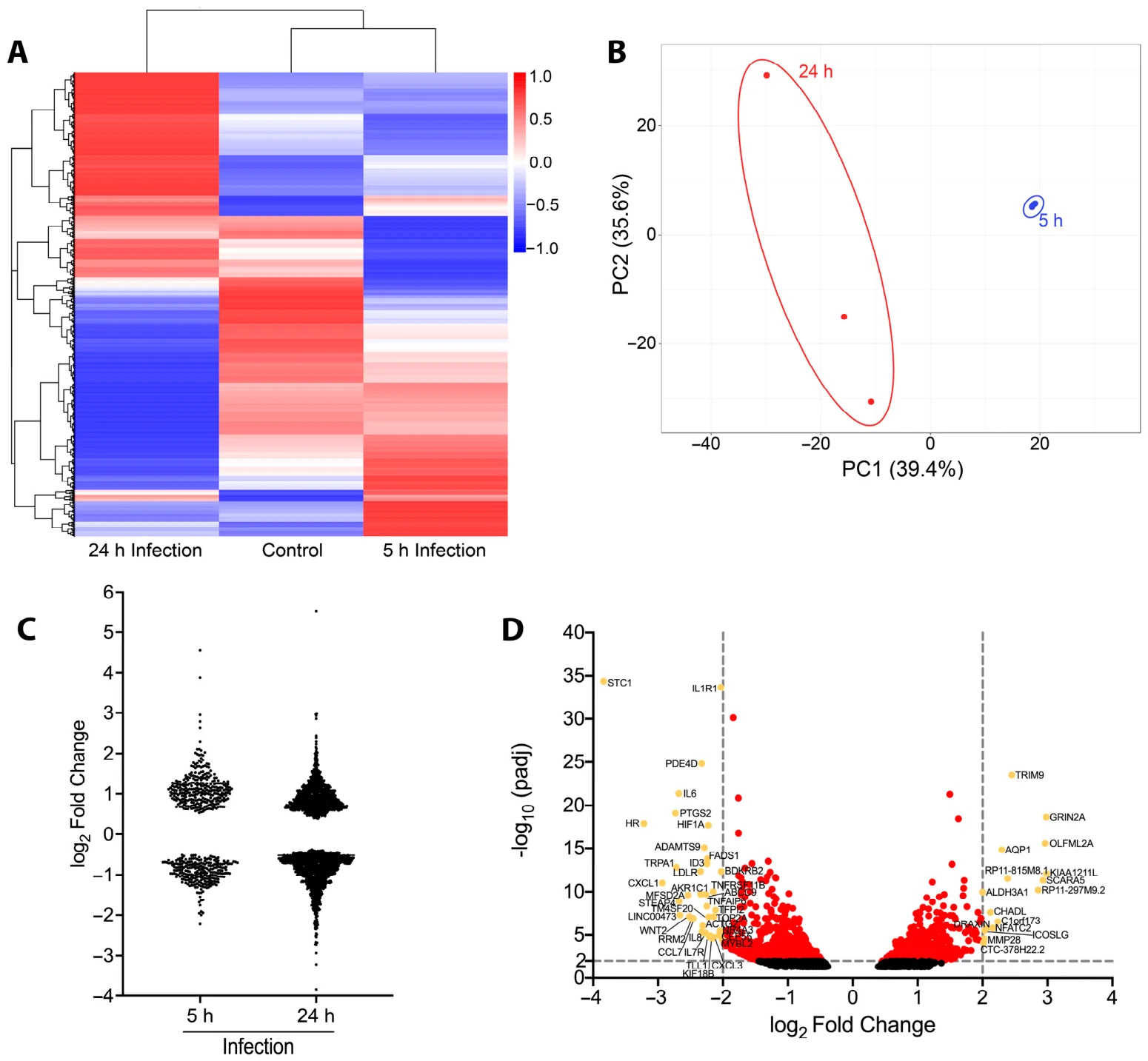
*T. denticola* alters the host’s gene expression of over 2000 genes within 24 h of infection. (**A**) Hierarchical clustering heatmap of differentially expressed genes across control, 5 h, and 24 h *T. denticola*-infected samples. Relative gene expression is displayed on a normalized scale ranging from −1 (blue, lower expression) to +1 (red, higher expression); reproduced from Ganther et al. [11] under the Creative Commons Attribution 4.0 International license (CC BY 4.0). (**B**) Principal component analysis (PCA) of the genes altered by *T. denticola* after 5 h and 24 h of infection. (**C**) Log_2_ fold change of the genes altered by *T. denticola* after 5 h and 24 h of infection, compared to control (no infection). (**D**) Volcano plot of the gene-expression changes caused by *T. denticola* invasion of hPDL cells compared to control. Red signifies genes with -log_10_ adjusted *p*-values > 2; Gold signifies genes with -log_10_ adjusted *p*-values > 2 and with Log_2_ fold change > 2. The highlighted genes were those that were the most significant altered by *T. denticola* infection. N = 1 independent biological replicate in triplicate.

To further evaluate the impact of *T. denticola* on hPDL cells, we conducted additional analyses of a previously published RNA-seq dataset generated from hPDL cells exposed to the bacterium for 5 h or 24 h (Figure 7). These data were previously generated by our group and initially analyzed by Ganther et al. [11] and deposited under doi.org/10.6084/m9.figshare.32652729.

Hierarchical clustering analysis of the resulting gene expression (Figure 7A) is reproduced from Ganther et al. [11] and is included here to provide context for the newly performed analyses. The 5 h and 24 h time points represent distinct stages of infection: 5 h corresponds to peak colocalization with the early endosomal marker Rab5, reflecting an earlier stage of bacterial internalization and host response, whereas 24 h represents an established intracellular bacterial burden characterized by predominant localization within late endosomal compartments and progression through lysosomal trafficking. The previously published clustering analysis showed that the gene-expression profile at 5 h of *T. denticola* infection clustered closely with that of the uninfected control, despite measurable transcriptional changes at this early time point. In contrast, the 24 h infection group formed a distinct cluster from both the control and 5 h groups, indicating a more pronounced alteration in the host gene-expression profile at the later stage of infection.

Building on these previously published findings, we performed a series of new analyses specifically for the present study to further characterize the temporal host transcriptional response to *T. denticola*.

Principal component analysis (PCA) was newly performed to evaluate global differences in gene-expression patterns between the experimental groups (Figure 7B). Together, the first two principal components explained 75% of the total variance, demonstrating that the two-dimensional PCA plot captured the majority of the variation within the dataset. The results reinforce the hierarchical clustering findings, showing a complete separation between the two time-point ellipses, with the 24 h cluster indicating a higher variance (larger ellipse), especially varying around principal component 2 (PC2) compared to the 5 h cluster, potentially emphasizing higher diversification or heterogeneity of host transcriptional responses during *T. denticola* invasion, especially at later stages. Together, these results suggest an early transcriptional host response to *T. denticola* invasion at 5 h, while 24 h infection is consistent with a broader shift to a distinct host transcriptional profile.

To further define the transcriptional changes occurring over time, a new analysis was performed by calculating log_2_ fold changes to directly compare gene expression at 5 h and 24 h after infection (Figure 7C). The results indicate that *T. denticola* significantly altered the expression of 560 genes within the first 5 h of infection and more than 2000 genes after 24 h.

A volcano plot was also newly generated to visualize the magnitude and statistical significance of differential gene expression at 24 h displaying log_2_ fold change versus -log_10_ adjusted *p* (padj) values (Figure 7D). Thresholds of ±2 log_2_ fold change (equivalent to a ≥4-fold change) and −log_10_(padj) > 2 (corresponding to a padj < 0.01) were applied and are indicated by the dashed lines in Figure 7D. Application of these criteria reduced the list of differentially expressed genes from more than 2000 to 52 high-confidence candidates.

Ganther et al. [11] have previously analyzed this RNA-seq dataset using Reactome and KEGG. In the present study, we performed a new analysis using the PANTHER Pathways classification system (Table 1). Reactome is a comprehensive pathway resource that focuses on detailed molecular reactions. It extends the concept of a reaction to encompass interactions involving proteins, nucleic acids, and small molecules [33]. KEGG represents genes, proteins, and small molecules as building blocks, which are interconnected by their interactions and reactions, thereby generating pathway maps of cellular and biological processes. PANTHER, on the other hand, is a comprehensive resource for the classification of genes according to their function and evolutionary history. For functional analyses, PANTHER Pathways uses Gene Ontology (GO) to provide one of the most comprehensive resources available regarding the functions of genes and gene products [34,35]. Because these resources differ in their annotation coverage, pathway definitions, and organizational structures, the PANTHER Pathways analysis provides a complementary perspective on the cellular processes affected by *T. denticola* and expands the functional interpretation of the dataset beyond the previously reported analyses.

Notably, the results show significant enrichment in the inflammation-mediated chemokine and cytokine signaling, and the p38 MAPK signaling pathways, as well as the p53 and apoptosis signaling pathways, suggesting overexpression of central regulators of periodontal inflammatory responses, cell survival, and tissue destruction mechanisms. Additional significant pathways included significant upregulation of integrin signaling, implicated in cell–matrix interactions and remodeling, and periodontal tissue integrity. Growth-factor signaling pathways, including significant enrichment of EGF, FGF, PDGF, and VEGF signaling, indicate overexpression of tissue repair and angiogenic responses. Genes involved in cholesterol biosynthesis were significantly upregulated following *T. denticola* infection, indicating altered host lipid metabolic activity potentially linked to membrane remodeling and intracellular signaling during infection. Collectively, these results indicate that *T. denticola* infection induces broad transcriptional changes affecting key biological processes involved in inflammation, host response regulation, and periodontal ligament cell homeostasis.

Finally, we hypothesized that nisin and SLN-Nisin may restore the host gene expression altered by *T. denticola* infection. To test this hypothesis, four genes, namely IL1R1, GRIN2A, STC1, and IL6, were selected from among the six most strongly differentially expressed genes identified by RNA-seq based on both the magnitude of their log_2_ fold changes and their −log_10_ adjusted *p*-values. The selected genes included both upregulated and downregulated targets and represented distinct biological functions affected by *T. denticola* infection.

Functionally, IL1R1 encodes interleukin-1 receptor type 1 (IL-1R1), which mediates pro-inflammatory signaling and plays an important role in the pathogenesis of periodontal disease [36]. IL6 encodes interleukin-6 (IL-6), a pleiotropic pro-inflammatory cytokine that promotes lymphocyte recruitment and contributes substantially to the host response to bacterial infection [37]. STC1 encodes a secreted protein implicated in angiogenesis–osteogenesis coupling by promoting angiogenesis and osteogenic differentiation and supporting type H blood vessel formation within the bone microenvironment [38]. GRIN2A encodes the GluN2A subunit, a component of a subset of NMDA glutamate receptors involved in calcium-dependent signaling. NMDA receptor-mediated glutamate signaling promotes the cytodifferentiation and mineralization of hPDL cells [39]. This suggests that dysregulation of STC1 and GRIN2A may affect PDL homeostasis and regenerative capacity.

**Figure 8:**
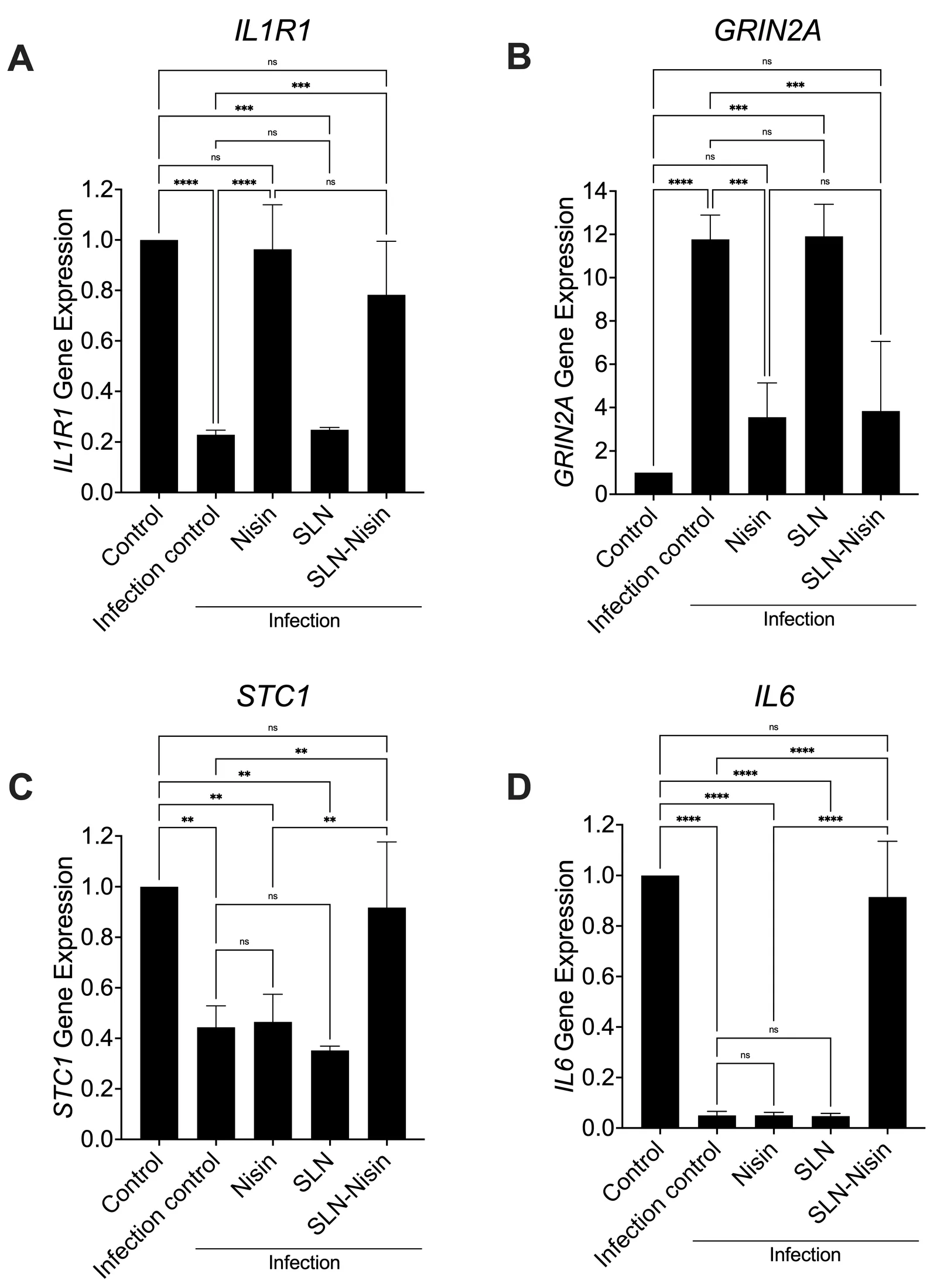
SLN-Nisin significantly restores *T. denticola*-mediated alterations in hPDL IL1R1 (**A**), GRIN2A (**B**), STC1 (**C**), and IL6 (**D**) gene expression towards control levels; validation via RT-qPCR. “ns” means not significant between marked samples, ** means *p* ≤ 0.01 between marked samples, *** means *p* ≤ 0.001 between marked samples, and **** means *p* ≤ 0.0001 between marked samples. N = 3 independent biological replicates (separate cell passages), each in duplicate.

Validation of the expression patterns of these four genes and responses to nisin and SLN-Nisin treatment were subsequently evaluated by RT-qPCR. The results (Figure 8) showed that *T. denticola* infection significantly increased GRIN2A levels, and significantly reduced IL1R1, STC1, and IL6 gene-expression levels compared to the control, thus validating our previous RNA-seq data. In terms of the treatments, two different patterns were observed in the data: 1) Both nisin and SLN-Nisin significantly recovered IL1R1 and GRIN2A expression toward control levels. For both genes, expression following either treatment was significantly different from the infection control but not from the uninfected control (Figure 8A and Figure 8B, respectively). No significant differences were observed between free nisin and SLN-Nisin for these genes. 2) Only SLN-Nisin significantly restored STC1 and IL6 expression toward control levels. Expression following SLN-Nisin treatment was significantly different compared to infection control but not from the uninfected control, whereas free nisin did not produce any significant recovery (Figure 8C and Figure 8D, respectively). Accordingly, SLN-Nisin differed significantly from free nisin for STC1 and IL6, but not for IL1R1 or GRIN2A. Together, these results indicate that both nisin and SLN-Nisin counteracted selected *T. denticola*-induced host gene-expression alterations, with SLN-Nisin restoring a broader set of evaluated genes compared to free nisin.

## 3. Discussion

In this in vitro proof-of-concept study, we demonstrate that *T. denticola* not only invades hPDL cells through endocytosis but also persists within late endosomal compartments for up to 24 h, inducing substantial host transcriptional alterations. SLN-Nisin is similarly internalized by hPDL cells, more specifically through both clathrin-mediated endocytosis and phagocytosis. Both nisin and SLN-Nisin significantly reduced bacterial burden and intracellular bacterial viability; notably, all three SLN-Nisin concentrations completely eliminated detectable intracellular *T. denticola*-positive hPDL cells and significantly reduced bacterial fluorescence intensity. Furthermore, SLN-Nisin exhibited a greater capacity to reverse the *T. denticola*-induced host transcriptional response, restoring four of the most strongly dysregulated genes to control levels compared with only two following nisin treatment.

These findings substantially expand our previous study [25], which demonstrated that SLN-Nisin inhibited the growth of planktonic *T. denticola* in axenic culture and disrupted saliva-derived oral biofilms. The present study advances this work by evaluating SLN-Nisin in a host-cell infection model and demonstrating its activity against intracellular *T. denticola*, and its capacity to restore selected pathogen-induced host gene-expression changes toward control levels.

Periodontal disease is a complex bacterial-driven inflammatory disease. A consortia of bacteria that include *T. denticola* are thought to act in synergy to subvert and overcome the host immune response [1]. Yet these microbes are also known to invade host cells to avoid the effects of antimicrobials, thereby causing persistent and recurrent infections [13,14]. Several groups have investigated invasion of periopathogens into gingival tissues, with *Fusobacterium nucleatum*, *Porphiromonas gingivalis, Aggregatibacter actinomycetemcomitans* and *T. denticola* being detected inside the gingival tissues and/or intracellularly [40,41,42,43]. In the case of *T. denticola*, Inagaki et al. [15] and Shin & Choi [16] demonstrated its intracellular invasion in human gingival epithelial cells, whereas Miao et al. [12] reported adherence to and internalization in hPDL cells. Bacterial invasion also requires the interaction of several molecules between bacteria and host cell, including Integrin β1 for *P. gingivalis* [44], and TLR2 and TLR4 for *T. denticola* [11,17,45]. In hPDL cells, bacterial adherence and internalization were observed by immunofluorescence microscopy, which also showed a close association between *T. denticola* and LAMP1-positive compartments. In a separate experiment, the authors assessed intracellular survival using an antibiotic protection assay followed by anaerobic culture of the hPDL-cell lysates. Viable *T. denticola* were recovered for up to 7 h but not after 24 h [12].

Our data demonstrated that *T. denticola* undergoes intracellular trafficking within host cells, with a subset of internalized bacteria being processed through the lysosomal pathway within 22 h, while another subpopulation persisted within late endosomal compartments for up to 24 h. Notably, we demonstrated that the bacterium remained viable after 24 h of invasion, supporting its capacity for intracellular persistence. It is possible that the use of solid media by Miao et al. [12], rather than broth-based culture, imposed additional selective pressure on the metabolically stressed *T. denticola*, thereby contributing to the apparent loss of viability observed in their study. Our data also showed that about 100% of hPDL cells were positive for *T. denticola* after 48 h, and 80% of the cells remained positive after 96 h of infection, further highlighting the potential persistence of the bacterium inside the host cell.

Inagaki et al. [15] demonstrated that a *T. denticola* dentilisin-deficient mutant had significantly lower invasion capabilities in gingival epithelial. Kamarajan et al. [17] showed similar results with TLR2 knockdown in hPDL cells and wild-type bacterium. These results indicate that dentilisin and TLR2 may have a pivotal role in the invasion process, with dentilisin potentially promoting the invasion of *T. denticola* via TLR2, with eventual processing of the bacterium via endocytosis. Yet, further studies are needed to fully understand the mechanism by which dentilisin interacts with TLR2 to promote host cell invasion.

The previous literature has shown that SLN-Nisin can improve nisin’s efficacy in inhibiting the growth of planktonic *T. denticola*, and in disrupting saliva-derived oral biofilms [25]. Our findings here extend these observations to intracellular *T. denticola*. All three SLN-Nisin concentrations produced significantly greater reductions in intracellular bacterial signal than free nisin at 26 µg/mL, with a near-complete absence of detectable *T. denticola* across the tested concentration range. Notably, SLN-Nisin remained more effective than free nisin even at the lowest nisin-equivalent concentration tested (6.5 µg/mL), which was fourfold lower than the free-nisin concentration. The absence of significant differences among the three SLN-Nisin groups suggests that the response reached a plateau within the tested range, possibly because 6.5 µg/mL was already sufficient to produce the maximum effect detectable under these experimental conditions. Kamarajan et al. [17] reported a comparable level of intracellular clearance using free nisin at 100 µg/mL. Although the experimental conditions differed between the two studies, which preclude a direct comparison of efficacy, it is noteworthy that a comparable effect was observed here with SLN-Nisin at a nisin-equivalent concentration of 6.5 µg/mL, an approximately 15-fold lower nisin-equivalent concentration. This supports that SLN-mediated delivery may enhance nisin activity against intracellular *T. denticola* and further highlights the advance provided by the present study. Additional dose–response studies incorporating concentrations below 6.5 µg/mL will be needed to define the minimum effective concentration of SLN-Nisin and quantify the potency increase.

Despite residual detection of *T. denticola* puncta by confocal microscopy after nisin treatment, no viable bacteria were recovered following host cell lysis. These findings suggest that nisin may have promoted *T. denticola*’s membrane damage and/or other cellular alterations that would compromise their viability, as evidenced by their inability to recover and re-establish growth following culture. In contrast, SLN-Nisin achieved greater intracellular bacterial clearance and reduced intracellular bacterial burden at substantially lower concentrations, highlighting the enhanced efficacy of the nanoparticle-based formulation. Nevertheless, the absence of a strong fluorescent signal (*T. denticola* puncta) does not necessarily indicate the complete physical removal of all bacterial genetic material. The residual low-level fluorescence observed indicates the presence of non-viable bacterial fragments or that bacterial genetic material still remains within the host cells, which correlates with the observed loss of bacterial viability.

Our RNA-seq results also indicate that, once inside, *T. denticola* significantly alters the gene expression over 2000 genes in hPDL cells within 24 h, highlighting that the bacterium can quickly subvert and compromise hPDL cell gene expression. To confirm the robustness of this transcriptomic analysis, we validated four representatives differentially expressed genes by RT-qPCR. Gene selection was based on stringent statistical significance, substantial changes in expression, and relevance to periodontal disease biology.

The validation panel included both upregulated and downregulated transcripts, as well as biological relevance to periodontal disease. IL6 and IL1R1 represent well-established mediators of pro-inflammation, collagen destruction, and bone resorption in periodontal disease and are usually found to be significantly increased in patients with periodontal disease [37,46]. Our data highlights a reduced expression of this interleukin gene at 24 h of infection. However, in the context of established intracellular persistence of *T. denticola*’s in the late endosome at 24 h, the reduced expression of IL6 together with IL1R1 may reflect active modulation of host inflammatory signaling by *T. denticola* at 24 h rather than an absence of inflammation. This downregulation may, consequently, diminish the capacity of infected hPDL cells to sense and propagate pro-inflammatory signals and may represent a strategy by which *T. denticola* promotes intracellular persistence, enabling survival within host cells. Long-term effects of reductions in IL6 and IL1R1 expression could impair IL-6 production and cellular responsiveness to IL-1, respectively, potentially compromising host defense. For example, in a murine model of pneumococcal pneumonia, IL-6-deficient mice exhibited significantly increased pulmonary levels of pro-inflammatory cytokines, including TNF-α, IL-1β, and IFN-γ, together with significantly higher *Streptococcus pneumoniae* burdens and earlier mortality than infected wild-type mice [47]. Although these findings cannot be directly extrapolated to periodontal infection, they highlight the protective role of these cytokines signaling during bacterial infection. Thus, sustained reduction in IL6 and IL1R1 expression may impair bacterial control and promote dysregulated inflammation, potentially contributing to further periodontal tissue destruction. In this regard, more studies are warranted to determine how these sustained alterations in IL6 and IL1R1 expression would affect periodontal host defense, bacterial persistence, and tissue homeostasis.

STC1 and GRIN2A represent potentially novel pathways involved in host responses to *T. denticola* infection. In particular, STC1 encodes for a glycoprotein expressed in a variety of tissues, with multiple autocrine and paracrine functions related to calcium and phosphate influx and tissue homeostasis. In periodontal ligament tissues, STC1 has been associated in bone remodeling and promoting angiogenesis and osteogenic differentiation, while supporting type H blood vessel formation within the bone microenvironment [38,48]. Conversely, GRIN2A encodes for the GluN2A subunit of the N-methyl-D-aspartate (NMDA) receptor, a glutamate receptor, which also regulates calcium influx. Fujihara et al. [39] have previously demonstrated that the NMDA receptor is present in PDL cells and its activation promotes PDL cell cytodifferentiation and mineralization during physiological mechanical loading through calcium-dependent intracellular signaling. Although the biological significance of GRIN2A and STC1 expression during *T. denticola* invasion remains to be determined, it may suggest that intracellular infection may disrupt calcium-dependent signaling pathways involved in periodontal ligament homeostasis. These findings together indicate the possibility that, beyond modulating inflammatory responses, *T. denticola* may alter cellular programs governing tissue remodeling and maintenance, although the functional consequences of these transcriptional changes require further investigation. Together, these findings provide additional insight into the molecular mechanisms underlying *T. denticola*-mediated pathogenesis.

SLN-Nisin significantly reversed these four *T. denticola*-induced altered genes toward control levels, whereas nisin was able to revert only two of these. Taken together, the lower intracellular bacterial burden and greater recovery of selected host gene-expression responses toward control levels suggest that SLN-Nisin may exert broader biological effects than free nisin. By simultaneously targeting the pathogen and promoting recovery of the host response toward control levels, SLN-Nisin may represent a shift from conventional pathogen elimination-focused therapies toward strategies that also restore periodontal tissue homeostasis. Based on these findings and previous evidence of the beneficial effects of free nisin in in vivo models of periodontal disease [49], we hypothesize that, in an in vivo model of periodontal disease, SLN-Nisin could reduce the periodontal bacterial burden while promoting the recovery of pathogen-induced host gene-expression changes toward homeostatic levels. These combined effects may help regulate inflammation, limit periodontal tissue destruction, and promote the restoration of periodontal homeostasis more effectively than free nisin alone. In this context, these proof-of-principle findings provide a foundation for future transcriptomic and in vivo studies to evaluate the broader biological relevance and therapeutic potential of SLN-Nisin, compared with free nisin, in periodontal disease.

Both nisin [49,50,51,52,53] and SLN-based delivery systems [54,55,56,57] have previously demonstrated favorable safety and biocompatibility profiles in both in vitro and in vivo studies. In particular, Kamarajan et al. [52] reported no significant cytotoxic effects of nisin at concentrations up to 400 µg/mL in human oral keratinocytes in vitro or at doses up to 800 mg/kg/day in a mouse floor-of-mouth oral cancer xenograft model. Furthermore, nisin is recognized by the US Food and Drug Administration (FDA) as Generally Recognized as Safe (GRAS) [50], while the European Food Safety Authority (EFSA) established an acceptable daily intake of 1 mg nisin/kg body weight/day [51]. For the lipid nanoparticle, the literature indicates that most evaluated cell lines tolerate lipid concentrations of up to 1 mg/mL in SLN formulations [54]. Importantly, our previous study testing this particular SLN formulation determined an IC_50_ of 0.065 mg/mL of lipids for SLNs in BALB/c 3T3 fibroblasts, a cell line very sensitive to cytotoxic effects [55]. At the highest nisin-equivalent concentration tested here (26 µg/mL), SLN-Nisin contained an estimated lipid concentration of approximately 0.03 mg/mL, which is approximately 2.2-fold lower than the IC_50_ in BALB/c 3T3 cells. Furthermore, a review of more than 30 in vivo studies found no major adverse effects associated with SLN administration, although mild alterations in some biochemical and hematological parameters were reported [54]. In particular, Mendonça et al. [56] specifically evaluated the unloaded SLN formulation used here in an in vivo model and observed only mild and transient effects, supporting its favorable preclinical safety profile as a potential nanoscale drug-delivery system. Nevertheless, nanoparticle safety may vary according to composition, dose, route of administration, and incorporated cargo. In this context, further studies evaluating the cytotoxicity and biocompatibility of SLN-Nisin in both in vitro and in vivo models, as well as its effects on the commensal oral microbiota, are warranted to support its safety assessment and potential clinical translation.

The inhibition experiments indicate that SLN-Nisin uptake by hPDL cells involves both phagocytosis and clathrin-dependent pathways. While phagocytosis is commonly associated with the engulfment of comparatively larger particles (> 500 nm) [58,59,60], clathrin-mediated endocytosis has been reported to uptake beads up to 200 nm [61]. The SLN-Nisin formulation, with a mean diameter of 205 ± 7 nm, lies near this reported size range. Its internalization through both mechanisms indicate that particle size alone may not determine its cellular entry route for hPDL cells, and other physical–chemical characteristics may influence its uptake.

Following cellular uptake, nanoparticles can accumulate and cluster within intracellular vesicles through vesicle fusion and endosomal maturation [62]. For example, Liu et al. [62] demonstrated a gradual clustering of internalized gold nanoparticles during endosomal trafficking in HeLa cells. Larger clusters were found after nanoparticle entering the lysosomes and/or in the perinuclear region. Similar processes may explain the heterogeneous rhodamine-positive structures observed in the perinuclear region following SLN-Nisin treatment, particularly at nisin-equivalent concentrations of 13 and 26 µg/mL.

A potential mechanism underlying the enhanced activity of nanoparticle-associated nisin was investigated by Ratrey et al. [63]. Using molecular dynamic simulations, the authors reported that SLNs promoted the spatial support and confinement of nisin Z at the bacterial membrane interface and synergistically facilitated nisin binding to *Staphylococcus aureus* lipid II, a membrane-associated precursor of bacterial cell-wall synthesis [63]. Although their formulation and bacterial model differed from those used here, these findings suggest that a similar mechanism may underlie the particular SLN-Nisin formulation tested in this study. Yet, further studies are warranted to determine whether similar interactions contribute to this SLN-Nisin formulation against *T. denticola*, especially in the intracellular environment.

Limitations of this study include the use of a single bacterial species and a single host-cell type in an in vitro model and the lack of in vivo validation. Furthermore, periodontal disease is a complex condition involving synergistic interactions among multiple bacterial species within polymicrobial biofilms, together with dynamic responses from diverse host and immune cells [1]. Previous studies have shown that nisin significantly inhibits the growth of other periodontal pathogens, including *P. gingivalis*, *F. nucleatum*, and *A. actinomycetemcomitans*, in axenic culture [53,64]. However, higher nisin concentrations were required to completely inhibit these species compared to *T. denticola*. Based on the enhanced activity observed in the previous [25] and in the present studies, we hypothesize that SLN-mediated delivery may reduce the nisin concentrations required to inhibit these periodontal pathogens. Future studies using polymicrobial biofilms associated with multicellular host cells in vitro, as well as in vivo models are warranted to further evaluate SLN-Nisin’s efficacy.

Future studies would also benefit from determining whether the recovery of selected host gene-expression responses persists after nisin or SLN-Nisin withdrawal or if continuous treatment is required to maintain their levels. Time-course experiments evaluating the transcriptomic effect after treatment removal would assist defining the duration of the treatment activity post withdrawal. Given the greater activity of SLN-Nisin observed in the present study, we hypothesize that SLN-Nisin may permit either a shorter treatment duration at an equivalent nisin concentration or a similar treatment period over a lower nisin-equivalent concentration when compared with free nisin.

An additional limitation of this study is that the RNA-seq analysis was performed using a single biological replicate, albeit analyzed in technical triplicates. This design limits the estimation of biological variability of the transcriptome-wide findings. Nevertheless, the RT-qPCR validation supported the expression patterns of four selected genes identified in the RNA-seq dataset. Yet, additional RNA-seq studies incorporating at least three independent biological replicates of infected hPDL cells from three different passages are needed to further confirm the broader transcriptional response to *T. denticola*.

Although confocal microscopy enabled the quantitative assessment of intracellular *T. denticola* fluorescence while preserving spatial information, flow cytometry could provide a complementary, higher-throughput analysis across a larger population of hPDL cells. Future studies may employ flow cytometry to further extend the fluorescence-based quantification reported here.

These findings support SLNs as a promising delivery platform for AMPs, particularly nisin, and highlight the potential of SLN-Nisin as a therapeutic strategy for periodontal disease. By enabling intracellular delivery of nisin, SLN-Nisin may provide means to target periodontal pathogens that evade conventional antimicrobial treatment through invasion and persistence within host cells.

## 4. Materials and Methods

### 4.1. Ethics Statement

The University of California San Francisco Institutional Review Board (IRB) authorized the collection and use of periodontal ligament tissue from extracted teeth (#16–20204; reference #227030). The approved protocol indicated that the hPDL cells were exclusively isolated from clinically discarded teeth with intact roots during routine clinical care and showed no evidence of pathological changes or conditions. In this context, the IRB waived the requirement for patient informed consent, and no donor-identifying or demographic characteristics information was collected for this study.

### 4.2. Human-Derived Periodontal Ligament (hPDL) Cell Culture

Human-derived periodontal ligament (hPDL) was isolated from healthy human teeth collected, more specifically from the middle third of the roots, as described before [11,17]. Following isolation, fibroblasts outgrowths were cultured, in Minimum Eagle Medium-α (MEM-α) (Gibco, Grand Island, NY, USA) containing 10% fetal bovine serum (FBS; Gibco, USA), 1% penicillin/streptomycin (Gibco, USA), and 1% amphotericin B (Gibco, USA); cells were maintained in a Forma II incubator (Thermo-Fisher Scientific, Waltham, MA, USA) at 37 °C with a humid atmosphere containing 5% CO_2_ (UCLA, Los Angeles, CA, USA). Fibroblast cell outgrowths were evaluated/validated morphologically and passaged upon reaching approximately 90% confluence. Only hPDL cells between passages 3 and 7 were used in the following experiments to minimize the effects of cellular aging and senescence.

### 4.3. T. denticola Cell Culture

*T. denticola* (ATCC 35405, Manassas, VA, USA) was cultivated in Oral Treponeme Enrichment Broth (OTEB; Anaerobe Systems, Morgan Hill, CA, USA) within a Bactron300 anaerobic chamber (Sheldon Manufacturing, Cornelius, OR, USA) and kept at 37 °C. The chamber was maintained under positive pressure with an anaerobic air gas mixture containing 10% CO_2_, 5% H_2_, and N_2_ as balance (UCLA, USA). Residual oxygen was continually removed using a palladium-coated catalyst system, which was regenerated weekly by heating at 200 °C for 8 h. The bacterial cultures were routinely passaged every 5–7 days to maintain cell growth. Cells were subcultured 2–3 days before each experiment to ensure that the cells were in the logarithmic growth phase.

### 4.4. hPDL Cells Challenge with T. denticola

hPDL cells were infected with *T. denticola* as previously established [11,17]. A suspension of *T. denticola* was centrifuged at 5000 RPM for 10 min, after which the OTEB supernatant (Anaerobe Systems, USA) was discarded. The bacterial pellet was resuspended in PBS (Thermo-Fisher, USA) and the bacterial concentration (CFU/mL) was determined from the optical density (OD)/absorbance at 600 nm using a Spectramax M2 microplate spectrophotometer (Molecular Devices, San Jose, CA, USA). The bacterial suspension was incubated with Syto9 (1:1000 *v*/*v*; Thermo-Fisher, USA) for 15 min protected from light. Unbound dye was removed by washing the bacteria 3 times with PBS (Thermo-Fisher, USA). The labeled bacteria were subsequently added to hPDL cell cultures at 50 multiplicities of infection (MOI) in serum- and antibiotic-free MEM-α media (Gibco, USA). This MOI was selected based on previous published evidence which indicated that the *T. denticola* MOI elicits a robust hPDL cellular response in hPDL cells without detectable cytotoxicity [11]. The infected hPDL cells were maintained at 37 °C and 5% CO_2_ (UCLA, USA) for 2 h of incubation and then washed three times with PBS (Thermo-Fisher, USA) to remove any non-adherent bacteria.

### 4.5. Gentamicin Treatment to Remove Extracellular T. denticola

In order to detect only the intracellular bacteria and to kill/remove any lingering extracellular bacteria, post-infected hPDL cells were treated with gentamycin (Millipore-Sigma, Burlington, MA, USA), a bactericidal aminoglycoside antibiotic that is unable to penetrate mammalian cells [65]. Specifically, hPDL cells were treated with gentamicin (100 µg/mL) at 37 °C for 1 h after each *T. denticola* infection time-point or after nisin/SLN-Nisin treatment.

### 4.6. SLN and SLN-Nisin Synthesis

Our previously published method for microemulsion extrusion [25] was used to synthesize SLN. Stearic acid (7 mM, Millipore-Sigma, USA) was first heated to 75 °C until completely melted. DOTAP (2.5 mM; Avanti Polar Lipids, Alabaster, AL, USA), a quaternary cationic lipid, and Pluronic F68 (1 mM; Millipore-Sigma, USA), a nonionic surfactant, were then added while the mixture was maintained at 75 °C. After the lipid components had completely melted, the formulation was vortexed three times and passed through a 0.1 μm polycarbonate membrane using an extrusion apparatus maintained at 75 °C. The resulting SLN suspension was cooled in an ice bath and stored at 4 °C until use.

The nisin-loaded SLN (SLN-Nisin) synthesis method is a modification of this previous method [25]. To incorporate nisin into the SLN lipid matrix, nisin Z (Handary, Belgium) was dissolved in Milli-Q water (Millipore-Sigma, USA) at 5 mg/mL. The solution was mixed on a rotator for 4 h and passed through a 0.22 µm filter before nanoparticle synthesis. During nanoparticle synthesis, an appropriate volume of the filtered nisin Z solution (Handary, Belgium) was added to the lipid mixture maintained at 75 °C, immediately before extrusion, to achieve a final nisin concentration of 3.25 mg/mL in the SLN-Nisin formulation.

SLNs and SLN-Nisin were previously characterized by our group [25,55]. The unloaded SLN formulation had a mean diameter of 105 ± 1 nm and a polydispersity index (PDI) of 0.052 ± 0.013, whereas SLN-Nisin had an average diameter of 205 ± 7 nm and a PDI of 0.329 ± 0.04. SLN-Nisin also exhibited an encapsulation efficiency of 97.0 ± 1.1% of the initial nisin concentration of 3.2 mg/mL [25]. The morphology of both SLNs and SLN-Nisin was also characterized and reported previously [25,66]. In addition, the unloaded SLN formulation was previously shown to remain stable for up to 150 days when stored at 4 °C [55]. Before each use, both formulations were visually inspected for precipitation. Any batch exhibiting visible precipitation was discarded and replaced with a freshly prepared batch.

Fluorescent-labeled SLNs and SLN-Nisin were prepared by adding Rhodamine-PE (Avanti Polar Lipids, USA) to the lipid mixture before extrusion at a molar proportion of 0.4 mol% relative to the total lipid content.

### 4.7. Kinetic Assay for T. denticola Internalization

To test the ability of *T. denticola* to invade hPDL cells, hPDL cells (1 × 10^5^ cells/well) were seeded into 12-well plates (Corning, NY, USA) onto sterile 12 mm round coverslip slides (Fisher Scientific, USA) and allowed to adhere overnight. The next day, hPDL cells were washed in PBS (Thermo-Fisher, USA) and then challenged with Syto9-stained *T. denticola* for 2, 4, 6, 8, 10, 12 and 24 h in FBS- and antibiotics-free MEM-α media (Gibco, USA), followed by gentamicin treatment for 1 h, as described earlier. At the end of the experiment, cells were fixed with 4% paraformaldehyde (PFA; Thermo-Fisher, USA) for 20 min and then washed three times with PBS (Thermo-Fisher, USA). Next, the cells were incubated with Hoechst 333258 nuclear staining (1:1000; Millipore-Sigma, USA) for 30 min and then washed three times in PBS (Thermo-Fisher, USA). Finally, the coverslips were mounted to a slide using Dako Faramount (Agilent, Santa Clara, CA, USA) and the slides were imaged using an SP8 X Confocal Laser Scanning Microscope (Leica, Wetzlar, Germany). Captured images were processed using FIJI/ImageJ imaging (version 2.18.0) analysis software.

### 4.8. T. denticola Colocalization with Endocytosis Markers (Rab5, Rab7, and Lamp1)

To evaluate the *T. denticola* invasion pathway, hPDL cells (1 × 10^5^ cells/well) were seeded into 24-well plates (Corning, USA) onto sterile 12 mm round coverslip slides (Fisher Scientific, USA) and allowed to adhere overnight. The next day, the cells were transduced overnight with lentiviral vectors for either Rab5-RFP (CellLight Early endosomes-RFP; Thermo-Fisher, USA), Rab7-RFP (CellLight Late endosomes-GFP; Thermo-Fisher, USA), or Lamp1-RFP (CellLight Lysosome-RFP; Thermo-Fisher, USA) in FBS- and antibiotic-free MEM-α (Gibco, USA). Then, the transduced hPDL cells were challenged with Syto-9-stained bacteria, as described earlier: from 1 h to 6 h for the Rab5-RFP transduced cells, 8 h to 24 h for Rab7-RFP transduced cells, and 24 h to 28 h for Lamp1-RFP transduced cells. At the end of the experiment, cells were treated with gentamicin for 1 h, as described above, fixed with 4% PFA (Thermo-Fisher, USA) for 20 min, and washed three times with PBS (Thermo-Fisher, USA).

Next, the cells were incubated with Hoechst 333258 nuclear stain (1:1000; Millipore-Sigma, USA) for 30 min and then washed three times in PBS (Thermo-Fisher, USA). Finally, the coverslips were mounted to a slide using Dako Faramount (Agilent, USA) and the slides were imaged using an SP8 X Confocal Laser Scanning Microscope (Leica, Germany).

Captured images were analyzed for colocalization within hPDL cells using the JaCoP plugin in ImageJ/Fiji [28]. The red fluorescence channel (corresponding to the endocytic markers Rab5-RFP, Rab7-RFP, or LAMP1-RFP) and the green fluorescence channel (corresponding to Syto 9-labeled *T. denticola*), were imported separately into the plugin, and an intensity cutoff was applied to each channel to distinguish fluorescent objects from the background signal (threshold) before colocalization analysis. Object-based colocalization was then assessed by identifying discrete objects in the two images that spatially overlapped. Each overlap between a red-channel object and a green-channel object was considered one colocalization site, and the total number of such sites was quantified for each image. Pearson’s correlation coefficient was also calculated to assess the linear correlation between the fluorescence intensities of the red and green channels.

Pearson’s correlation coefficient ranges from −1 to +1 and was used to assess the linear relationship between fluorescence intensities in the two channels. Positive coefficients indicate a direct correlation between channel intensities, whereas negative coefficients indicate an inverse correlation. Values approaching +1 or −1 represent increasingly strong positive or negative correlations, respectively, while values near 0 indicate little to no linear correlation between the fluorescence channels [28,29]. For both analyses, five images per condition were used to capture the data, with results expressed as the mean ± SD to reflect image-to-image variability.

For pictures capturing both hPDL cell nuclei and *T. denticola* genetic material in the same channel (via Hoescht 333258 use; i.e., Figures S4 and S5 in the Supplemental Materials), hPDL nuclei were manually removed from the images before quantification of *T. denticola* fluorescence intensity.

### 4.9. Kinetic Assay for SLN-Nisin Internalization

The cellular uptake kinetics of SLN-Nisin was evaluated by flow cytometry, as previously published [67]. hPDL cells were seeded into 6-well plates (Corning, USA) at 1 × 10^5^ cells/well. After overnight incubation, the hPDL cells were subsequently washed in PBS (Thermo-Fisher, USA) and exposed to 13 µg/mL of nisin-equivalent Rhodamine-labeled SLN-Nisin in antibiotic-, serum-, and phenol red-free MEM-α (Thermo-Fisher, USA) for 0.5, 1, 2, 4, 6, and 8 h. At the end of each time point, the hPDL cells were rinsed with PBS (Thermo-Fisher, USA), trypsinized with phenol red-free 0.25% Trysin (Gibco, USA) for 5 min, and analyzed using a FACScalibur flow cytometer (BD, USA).

### 4.10. Inhibition of the SLN-Nisin Endocytosis Pathway

The contribution of the four main endocytosis pathways to SLN-Nisin internalization (namely, phagocytosis, clathrin-mediated, caveolin-mediated, and micropinocytosis) was determined by using endocytosis inhibitors, as previously published [67]. hPDL cells were seeded into 6-well plates (Corning, USA) at 1 × 10^6^ cells/well. After overnight incubation, the cells were then rinsed with PBS (Thermo-Fisher, USA) and pre-incubated for 30 min with one of the inhibitors in phenol red-free MEM- α (Gibco, USA). The inhibitor concentrations were selected based on previous publications [68]. A preliminary assessment of their effect on cell viability is provided in Table 2. Next, 13 µg/mL of nisin-equivalent Rhodamine-labeled SLN-Nisin was added to the inhibitor-containing medium. After 4 h of incubation, the hPDL cells were rinsed with PBS (Thermo-Fisher, USA), trypsinized with phenol red-free 0.25% Trysin (Gibco, USA) for 5 min, and analyzed using a FACScalibur flow cytometer (BD, Franklin Lakes, NJ, USA).

### 4.11. Inhibition of T. denticola Burden by SLN-Nisin

The effects of free nisin and SLN-Nisin on inhibiting established *T. denticola* burden were initially assessed by flow cytometry. hPDL cells were plated in 6-well plates (Corning, USA) at 3 × 10^6^ cells/well. After overnight incubation, the hPDL cells were infected with *T. denticola*, as described earlier, and exposed for 4 h to either free nisin at 26 µg/mL (Handary, Belgium) or SLN-Nisin at the same nisin-equivalent concentration. The hPDL cells were subsequently rinsed with PBS (Thermo-Fisher, USA), trypsinized with phenol red-free 0.25% Trysin (Gibco, USA) for 5 min, and analyzed using a FACScalibur flow cytometer (BD, USA).

To fully evaluate nisin and SLN-Nisin effects on burden, hPDL cells (1 × 10^5^ cells/well) were seeded into 24-well plates (Corning, USA) onto sterile 12 mm round coverslip slides (Fisher Scientific, USA) and allowed to adhere overnight. Then, the hPDL cells were challenged with Syto-9-stained *T. denticola*, as described earlier, for 24 h. Next, the infected cells were treated with either free nisin (26 µg/mL; Handary, Belgium) or Rhodamine-labeled SLN-Nisin at nisin-equivalent concentrations of 6.5, 13, or 26 µg/mL.

As described above, SLN-Nisin has a nisin encapsulation efficiency of 97.0 ± 1.1% [25], corresponding to an estimated encapsulated nisin concentration of 3.10 mg/mL. The SLN-Nisin formulation was then diluted based on this concentration to obtain nisin-equivalent concentrations of 6.5, 13, or 26 µg/mL. Thus, the 26 µg/mL SLN-Nisin group was directly matched to the nominal concentration of the free-nisin treatment. For the unloaded SLN control, a formulation volume equivalent to that administered in the 26 µg/mL SLN-Nisin group was used.

At the end of the experiment, cells were treated with gentamicin (Millipore-Sigma, USA, fixed with 4% PFA for 20 min and washed three times with PBS. The cells were then incubated with Hoechst 333258 nuclear staining (1:1000; Millipore-Sigma, USA) for 30 min and washed three times in PBS. Finally, the coverslips were mounted to a slide using Dako Faramount (Agilent, USA) and the slides were imaged using an SP8 X Confocal Laser Scanning Microscope (Leica, Germany). Captured images were analyzed using ImageJ/Fiji. The background was subtracted (20 pixels) in all green fluorescence images.

### 4.12. Assessment of Internalized T. denticola Survival

To assess the potential for *T. denticola* to invade hPDL cells and survive inside the cells, hPDL cells (5 × 10^5^ cells/well) were seeded onto 6-well plates (Corning, USA) and allowed to adhere overnight. The next day, the hPDL cells were challenged with *T. denticola* for 2 h, as described earlier, and treated with either free nisin (26 µg/mL; Handary, Belgium), SLN-Nisin (equivalent of 6.5, 13 or 26 µg/mL of nisin), or free SLNs (volume equivalent of 26 µg/mL of SLN-Nisin). After 24 h of infection, gentamicin treatment (1 h) was used to remove any extracellular *T. denticola*. The hPDL cells were then rinsed once with PBS (Thermo-Fisher, USA) and once with sterile milli-Q water (Millipore-Sigma, USA) to remove any traces of salt. Next, the hPDL cells were cytolyzed by incubating them with sterile milli-Q water (Millipore-Sigma, USA) for 1 h at room temperature and monitored under a microscope. Bacterial cells are known to resist cytolysis due to the presence of a cell wall, which does not exist in mammalian cells [69]; this property was leveraged to differentially cytolyze the hPDL cells but not the *T. denticola* bacteria. Further, given that *T. denticola* does not grow well in solid media, the cytolyzed cell remnants were scraped, collected, and added to 5 mL of OTEB media (1:5, *v/v* respectively; Anaerobe Systems, USA) and incubated in an anaerobic environment at 37 °C for 10 days in a Bactron300 anaerobic chamber (Sheldon Manufacturing, USA) to assess the growth of any *T. denticola* that may have been internalized. Finally, *T. denticola* growth was evaluated via spectrophotometry using a Spectramax M2 microplate spectrophotometer (Molecular Devices, USA) at OD_600_.

### 4.13. RNA-Sequencing (RNA-Seq) of T. denticola-Infected hPDL Cells

RNA sequencing (RNA-Seq) was used to assess the degree to which *T. denticola* alters hPDL cell gene expression, as previously published by Ganther et al. [11]. hPDL cells (9.5 × 10^5^ cells/well) were left either uninfected (control) or challenged with *T. denticola* (as described above) for either 5 h or 24 h. The experiment included one independent biological replicate (N = 1), with three technical replicates per condition. Then, RNA from the samples was extracted using an RNeasy Mini Kit (Qiagen, Hilden, Germany) according to manufacturer’s instructions. The quality of the extracted RNA was assessed using a Nanodrop UV–Vis Spectrophotometer (Thermo-Fisher Scientific, USA).

RNA-seq library preparation and paired-end sequencing were performed by Novogene Corporation (Sacramento, CA, USA).

Messenger RNA (mRNA) was enriched from total RNA using magnetic beads conjugated to poly-T oligonucleotide and subsequently fragmented. cDNA library was then prepared using the NEBNext Ultra RNA Library Prep Kit for Illumina (NEB, Ipswich, MA, USA), following the manufacturer’s instructions. In brief, first-strand cDNA was generated using random hexamer primers and M-MuLV Reverse Transcriptase (RNase H−), followed by second-strand synthesis using DNA Polymerase I and RNase H. The resulting double-stranded cDNA was purified with AMPure XP beads (Beckman Coulter, Indianapolis, IN, USA), end-repaired, adenylated at the 3′ ends, and ligated to NEBNext adaptors. Fragments containing inserts of approximately 150–200 bp were selected and amplified by PCR. Library concentration and quality were evaluated using a Qubit 2.0 fluorometer (Thermo-Fisher, USA) and an Agilent 2100 Bioanalyzer (USA). The libraries were then sequenced on a HiSeq 4000 platform (Illumina, San Diego, CA, USA) to generate 150 bp paired-end reads.

Raw sequencing reads were filtered by removing reads containing adaptor sequences, along with reads with more than 10% undetermined bases, and reads for which over 50% of the bases had a Phred quality score of 5 or lower. The resulting high-quality reads were aligned to the reference genome using STAR.

Previous downstream analysis of this RNA-seq dataset by Ganther et al. [11] included published hierarchical clustering and heatmap visualization of gene-expression profiles from uninfected control cells and cells infected with *T. denticola* for 5 h or 24 h. The previously published heatmap was included here to provide context for the newly performed analyses. For the present study, the dataset was further evaluated using principal component analysis (PCA), differential expression analysis, volcano plot representation, and pathway-enrichment analysis. PCA was performed using normalized expression values relative to uninfected controls to visualize overall differences in global gene-expression profiles between 5 h and 24 h, complementing the clustering analysis. Temporal changes in the magnitude of gene expression were assessed by the differential expression analysis. This calculates log_2_ fold changes separately for the 5 h and 24 h infection groups relative to the uninfected control. A volcano plot was generated focusing on the 24 h dataset to integrate the magnitude and statistical significance of the expression changes and to identify highly dysregulated candidate genes. Finally, differentially expressed genes were evaluated for their biological processes gene ontology using the PANTHER pathways (https://geneontology.org) with Homo Sapiens as reference organism [70].

### 4.14. hPDL Gene-Expression Changes Mitigated by Nisin and SLN-Nisin

The capacity of nisin and SLN-Nisin to restore selected infection-induced host gene-expression alterations was evaluated by RT-qPCR. hPDL cells were plated in 6-well plates (Corning, USA) at 5 × 10^5^ cells/well. After overnight incubation, the culture was infected with *T. denticola* for 24 h, using the procedure described above, followed by treatment with either nisin Z (Handary, Belgium) at 26 µg/mL or SLN-Nisin at 26 µg/mL of nisin-equivalent concentration. After an additional 24 h of incubation, total RNA was isolated from the hPDL cells using an RNeasy Micro Kit (Qiagen, USA) according to the kit’s instructions. A complementary DNA (cDNA) library was subsequently synthesized using a SuperScript™ VILO IV™ cDNA Synthesis Kit (Thermo-Fisher, USA) according to the kit’s instructions using a Quantstudio 3 (Thermo-Fisher, USA) instrument. Four of the top six genes altered by *T. denticola* (by log_2_-fold change and -log_10_ adjusted *p*-value; i.e., IL1R1, GRIN2A, STC1, and IL6, as indicated by the volcano plot (Figure 6D)) were then selected and tested via qPCR. The analysis included three independent biological replicates (N = 3), each derived from a separate hPDL cell passage and analyzed in technical duplicate. For each sample, 100 ng of cDNA template was added to a 20 µL reaction containing TaqMan™ Fast Advanced Master Mix (Thermo Fisher, USA). Multiplex qPCR was performed to simultaneously detect one target gene using a FAM-labeled probe and the housekeeping reference gene GAPDH using a VIC-labeled probe. The following commercially available TaqMan™ Gene Expression Assays (Thermo-Fisher, USA) were used: IL1R1 (Hs00991010_m1), GRIN2A (Hs00168219_m1), STC1 (Hs00174970_m1), IL6 (Hs00174131_m1), and GAPDH (Hs02786624_g1). Reactions were performed on a QuantStudio™ 3 Real-Time PCR System (Applied Biosystems, USA) according to the TaqMan™ manufacturer’s standard cycling protocol. Relative gene expression to control was calculated using the 2^−ΔΔCt^ method.

### 4.15. Statistical Analysis

All data was analyzed using GraphPad Prism software (version 11.1.0, GraphPad, USA) and the results were evaluated by one-way ANOVA and displayed as mean ± SD. For the RNA-Seq data, PCA analysis was performed via the web tool ClustVis [71], while the volcano plot analysis was performed using RStudio Software (version 2026.07, RStudio, Vienna, Austria).

## 5. Conclusions

Collectively, these findings demonstrate that SLN-Nisin is an effective intracellular delivery strategy that reduces both *T. denticola* invasion and persistence, thereby attenuating host transcriptional reprogramming and achieving a more complete restoration of cellular homeostasis compared to free nisin. This innovative approach may provide a promising strategy to enhance the treatment of periodontal disease and its associated systemic complications by improving the targeting and clearance of intracellular pathogens.

## Figures and Tables

**Table 1:** Dysregulation of cholesterol biosynthesis, inflammatory and apoptotic signaling pathways in hPDL cells in response to *T. denticola* infection.

PANTHER Pathways	Representation	Over/Under	Fold-Change	*p*-Value (−log10)	FDR (−log10)
Cholesterol biosynthesis	11/13	+	4.29	6.04	4.32
Circadian clock system	6/10	+	3.04	2.23	1.33
Alpha adrenergic receptor signaling pathway	11/24	+	2.33	2.48	1.48
p38 MAPK pathway	18/41	+	2.23	3.32	2.11
Insulin/IGF pathway–mitogen-activated protein kinase kinase/MAP kinase cascade	14/33	+	2.15	2.51	1.48
Integrin signaling pathway	76/192	+	2.01	9.71	7.80
Apoptosis signaling pathway	45/117	+	1.95	5.49	3.99
VEGF signaling pathway	25/68	+	1.87	2.97	1.88
Ubiquitin proteasome pathway	21/58	+	1.84	2.38	1.43
Angiogenesis	60/169	+	1.80	5.86	4.26
p53 pathway	31/88	+	1.79	3.19	2.02
EGF receptor signaling pathway	47/134	+	1.78	4.56	3.20
CCKR signaling map	60/173	+	1.76	5.33	3.91
Huntington disease	47/146	+	1.63	3.47	2.17
FGF signaling pathway	39/122	+	1.62	2.9	1.84
PDGF signaling pathway	45/144	+	1.59	3.00	1.87
Gonadotropin-releasing hormone receptor pathway	68/231	+	1.49	3.36	2.11
Inflammation mediated by chemokine and cytokine signaling pathway	71/262	+	1.38	2.43	1.46
Unclassified	3361/18,000	-	0.95	20.83	18.62
Heterotrimeric G-protein signaling pathway	1/37	-	0.14	2.23	1.31

**Table 2:** Endocytosis inhibitors (Millipore-Sigma, Burlington, MA, USA) used to evaluate SLN-Nisin endocytosis pathways, based on previous publications [68], and preliminary assessment of their effect on cell viability.

Compound	Endocytosis Pathway Inhibited	Concentration (µg/mL)
Cytochalasin D	Phagocytosis	5
Chlorpromazine	Clathrin-mediated	1
Methyl-B-Cyclodextrin	Caveolin-mediated	12.5
Wortmannin	Macropinocytosis	13

## Data Availability

The data presented in this study are openly available in [FigShare] at [doi.org/10.6084/m9.figshare.32652729].

## Supplementary Materials

The following supporting information can be downloaded at https://www.mdpi.com/article/10.3390/ph19101585/s1, Movie S1. 3D reconstruction of *T. denticola*-invaded hPDL cell v1; Movie S2. 3D reconstruction of *T. denticola*-invaded hPDL cell v2; Figure S1. SLN-Nisin undergoes internalization into periodontal ligament cells via endocytic mechanisms; Figure S2. Nisin and SLN-Nisin reduces *T. denticola* burden into hPDL cells after 4 h of treatment; Figure S3. SLN-Nisin colocalizes with Rab7 and *T. denticola* between 9 h and 10 h after infection; Figure S4. SLN-Nisin reduces intracellular and extracellular *T. denticola* after 48 h; Figure S5. SLN-Nisin reduces intracellular and extracellular *T. denticola* after 96 h.

## Abbreviations

The following abbreviations are used in this manuscript (in alphabetical order):

AMPs	Antimicrobial Peptides
ANOVA	Analysis of Variance
ATCC	American Type Culture Collection
CFU/mL	Colony Formation Units per milliliter
DOTAP	1,2-dioleoyl-3-trimethylammonium-propane
FBS	Fetal Bovine Serum
GRIN2A	Glutamate Ionotropic Receptor NMDA type subunit 2A
hPDL	Human Periodontal Ligament cells
IL1R1	Interleukin 1 receptor type 1
IL6	Interleukin 6
Lamp1	Lysosome-associated membrane protein 1
MEM- α	Minimum Essential Medium- α
MMP-2	Matrix Metalloproteinase-2
MOI	Multiplicities of Infection
Nano-DDSs	Nano-sized Drug Delivery Systems
NHANES	National Health and Nutrition Examination Survey
OD_600_	Optical Density/Absorbance at 600 nm
OTEB	Oral Treponeme Enrichment Broth
PBS	Phosphate Buffer Saline
PCA	Principal Component Analysis
PFA	Paraformaldehyde
Rab5	Ras-related protein 5
Rab7	Ras-related protein 7
RFP	Red-Fluorescent Protein
RPM	Rotations per Minute
SLNs	Solid Lipid Nanoparticles
SLN-nisin	Nisin-loaded Solid Lipid Nanoparticles
STC1	Stanniocalcin 1
*T. denticola*	*Treponema denticola*
TLR2	Toll-like Receptor 2
