# Supplementary material for: Nisin-Loaded Solid Lipid Nanoparticles (SLN-Nisin) Mitigate Treponema denticola Intracellular Infection and Restore Highly Dysregulated Host Gene Expression
Source: Pharmaceuticals (Basel). 2026 Oct 7;19(10):1585. doi: 10.3390/ph19101585 (PMC13708361; doi:10.3390/ph19101585)
Supplement: Supplementary file 1 [file pharmaceuticals-19-01585-s001.zip › Supplemental Mateirals.pdf]

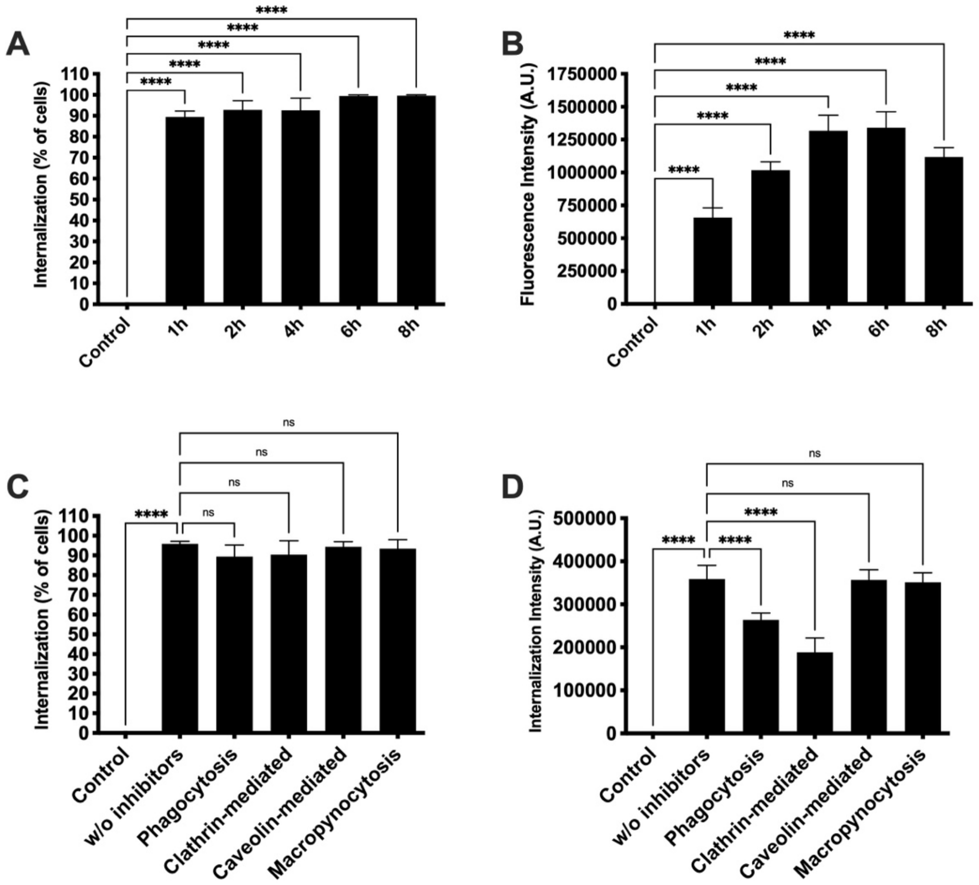

Figure S1. SLN-Nisin undergoes internalization into periodontal ligament cells via endocytic mechanisms. Flow Cytometry results for (A) Quantification of positive hPDL cells for SLN-Nisin internalization over time. (B) Fluorescence intensity of internalized SLN-Nisin over time. (C) Quantification of positive hPDL cells for SLN-Nisin internalization with or without inhibitors for phagocytosis, clathrin-mediated endocytosis, caveolin-mediated endocytosis and macropinocytosis. (D) Fluorescence intensity of internalized SLN-Nisin with or without inhibitors for phagocytosis, clathrin-mediated endocytosis, caveolin-mediated endocytosis and macropinocytosis. **** means $p \leq 0.0001$ between marked samples. N=3 independent biological replicates (separate cell passages) in triplicate

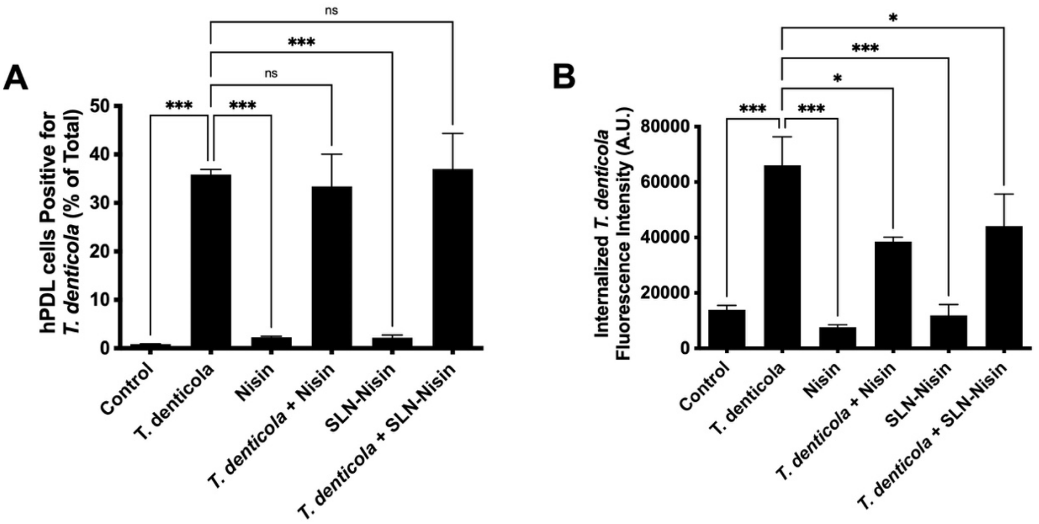

Figure S2 – Nisin and SLN-Nisin reduces *T. denticola* invasion into hPDL cells after 4h of treatment. (A) Quantification of *T. denticola*-positive hPDL cells treated with either nisin (26µg/mL) or SLN-Nisin (26µg/mL). (B) Fluorescence intensity of *T. denticola* in hPDL cells treated with either nisin (26µg/mL) or SLN-Nisin (26µg/mL). * Means p ≤ 0.05 between marked samples. *** means p ≤ 0.001 between marked samples. N=3 independent biological replicates (separate cell passages) in duplicate

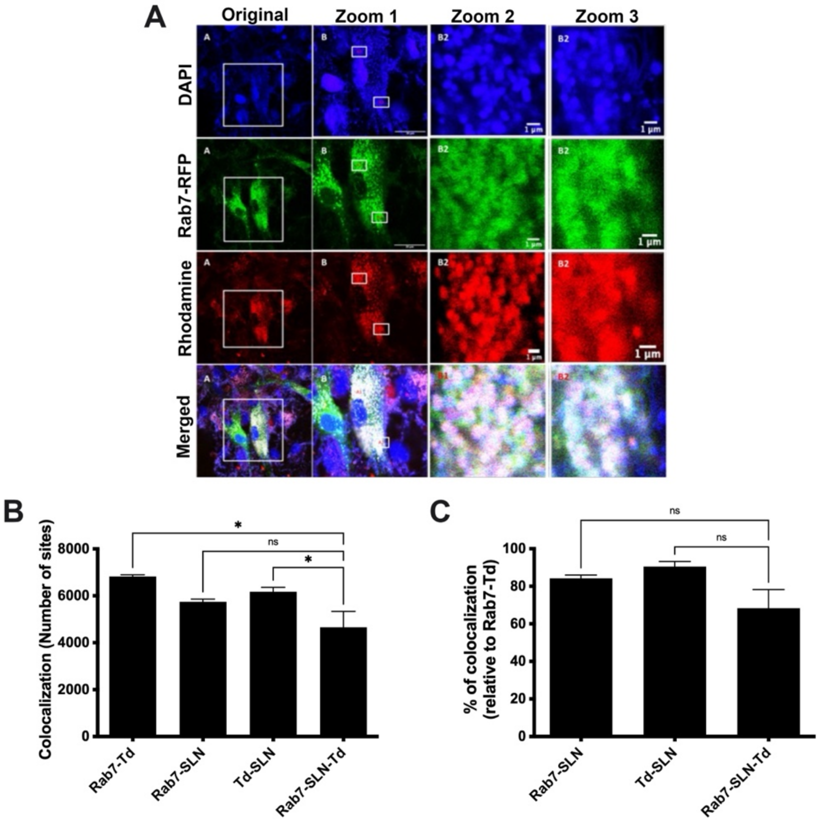

Figure S3 – SLN-Nisin colocalizes with Rab7 and *T. denticola* between 9h and 10h after infection. (A) Confocal microscopy of *T. denticola* (Top row; DAPI; Blue), hPDL Rab7 (Middle-upper row; Green),

SLN-Nisin (Middle-lower row; Red) and the merged image (bottom row). (B) Number of colocalizations in the images. (C) Percentage of colocalizations relative to Rab7 and *T. denticola*. * Means $p \leq 0.05$ between marked samples. N=3 independent biological replicates (separate cell passages) in duplicate

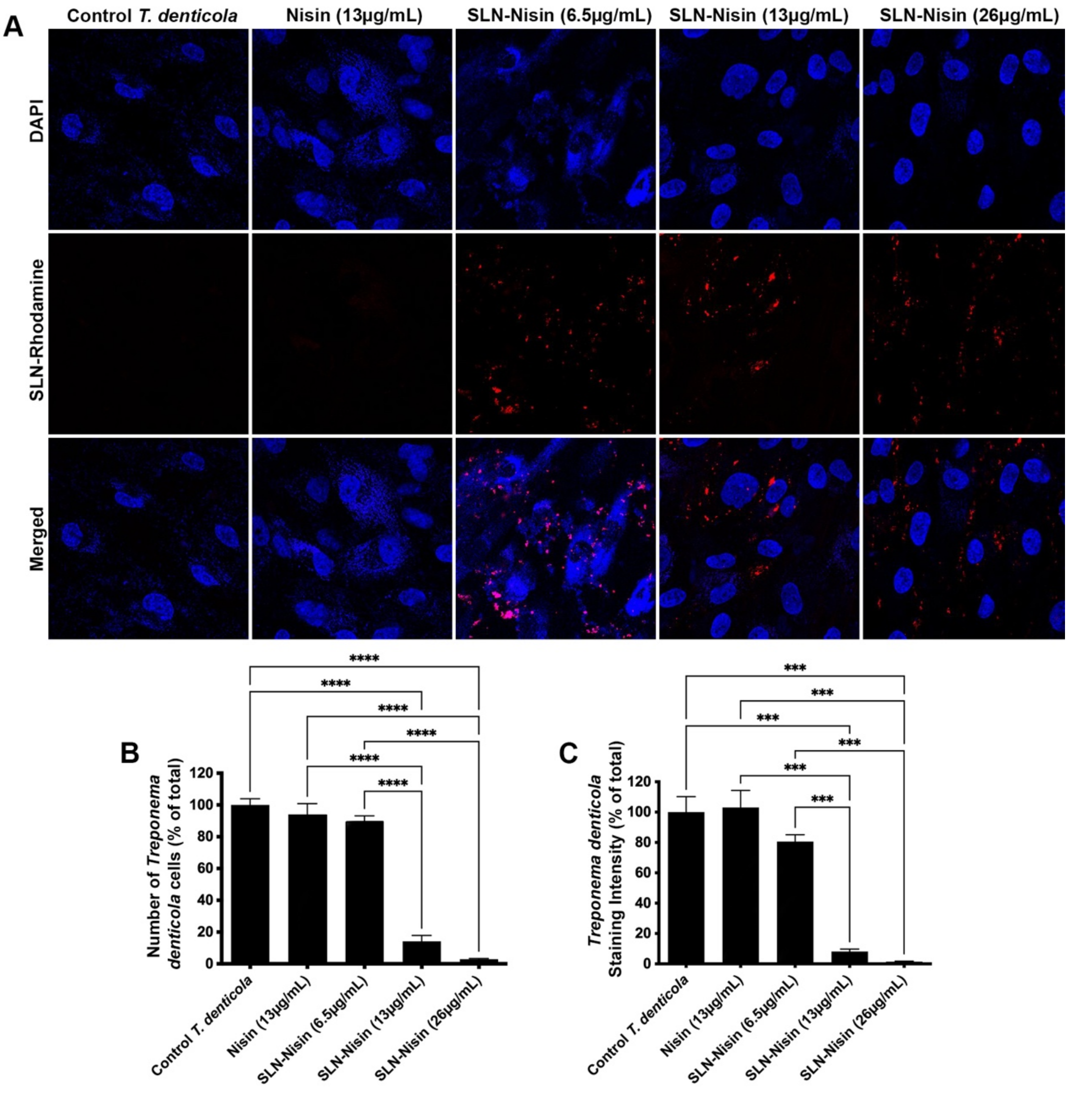

Figure S4. SLN-Nisin reduces intracellular and extracellular *T. denticola* after 48h. (A) Confocal microscopy of *T. denticola* genetic material (Top row; DAPI; Blue), SLN-Nisin (Middle row; Red) and the merged image (bottom row). (B) Number of *T. denticola* puncta in the images relative to control. (C) Percentage of fluorescence intensity relative to control (*T. denticola* infection) *** means $p \leq 0.001$ between marked samples and **** means $p \leq 0.0001$ between marked samples. N=2 independent biological replicates (separate cell passages) in duplicate.

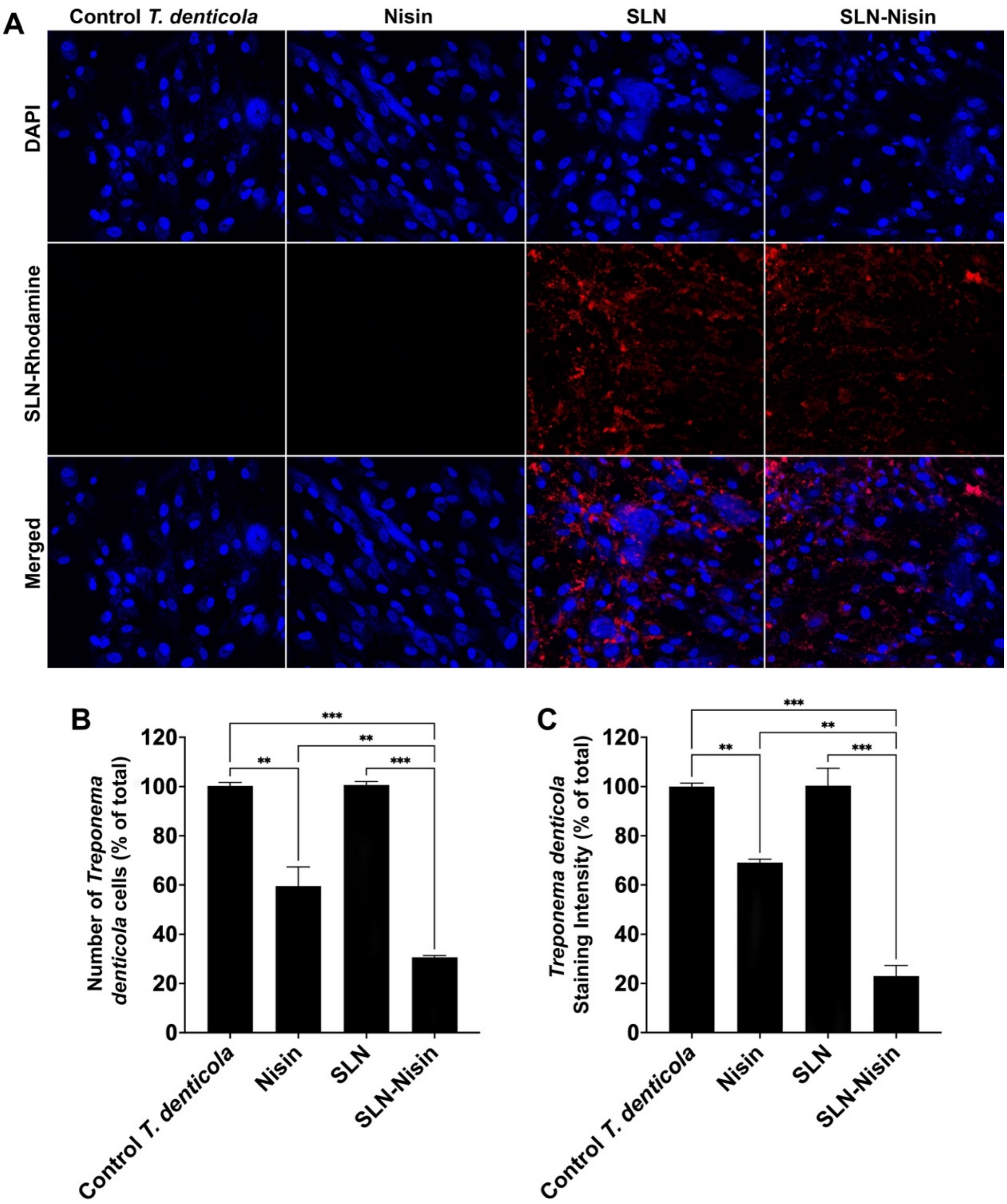

Figure S5. SLN-Nisin reduces intracellular and extracellular *T. denticola* after 96h. (A) Confocal microscopy of *T. denticola* and PDL cells genetic material (Top row; DAPI; Blue), SLN or SLN-Nisin (13µg/mL; Middle row; Red) and the merged image (bottom row). Nisin equivalent to 13µg/mL. (B) Number of *T. denticola* puncta in the images relative to control. (C) *T. denticola* fluorescence intensity relative to control. ** means $p \leq 0.01$ between marked samples, *** means $p \leq 0.001$ between marked samples and **** means $p \leq 0.0001$ between marked samples. N=2 independent biological replicates (separate cell passages) in duplicate.
